# Prevalence of asymptomatic *Leishmania* infection and knowledge, perceptions, and practices in blood donors in mainland Portugal

**DOI:** 10.1186/s13071-023-05980-1

**Published:** 2023-10-10

**Authors:** Rafael Rocha, Luzia Gonçalves, Cláudia Conceição, Patrícia Andrade, José Manuel Cristóvão, Jorge Condeço, Beatriz Delgado, Cristina Caeiro, Tetyana Kuzmenko, Eugénia Vasconcelos, Maria Antónia Escoval, Carmen Rey, Madalina Guz, Cláudia Norte, Carlos Aldeia, Diego Cruz, Carla Maia

**Affiliations:** 1 Instituto de Higiene E Medicina Tropical (IHMT), Universidade Nova de Lisboa (UNL), Lisbon, Portugal; 2 Global Health and Tropical Medicine (GHTM), Associate Laboratory in Translation and Innovation Towards Global Health, LA-REAL, IHMT, UNL, Lisbon, Portugal; 3 Centro de Estatística E Aplicações da Universidade de Lisboa (UL), Lisbon, Portugal; 4 Z-Stat4life, Lisbon, Portugal; 5 Instituto Português Do Sangue E da Transplantação, Lisbon, Portugal; 6 Centro Hospitalar Universitário Do Algarve, Portimão, Portugal; 7 Hospital do Espírito Santo de Évora, Évora, Portugal; 8 Unidade Local de Saúde Do Baixo Alentejo, Beja, Portugal; 9 Unidade Local de Saúde Do Litoral Alentejano, Santiago Do Cacém, Portugal; 10 Unidade Local de Saúde Do Norte Alentejano, Elvas, Portugal

**Keywords:** *Leishmania*, Leishmaniasis, Asymptomatic, Seroprevalence, Blood donor, Knowledge, Perceptions, Practices, Portugal, One Health

## Abstract

**Background:**

Asymptomatic infection is the most common outcome of exposure to *Leishmania* parasites. In the Mediterranean region, where *Leishmania infantum* is endemic, studies on the prevalence of asymptomatic infection have often relied on serological testing in blood donors. In Spain, regional studies have shown seroprevalence in blood donors between 1 and 8%; in Portugal, values of 0 and 2% were suggested by two localized studies, in different populations. The purpose of this study was (i) to estimate the prevalence of asymptomatic *Leishmania* infection in blood donors in mainland Portugal, and (ii) to study the association between the detection of antibodies to *Leishmania* and sociodemographic factors, and also the knowledge, perceptions and practices (KPP) of the blood donors regarding leishmaniasis.

**Methods:**

A cross-sectional study targeted the population of people who donated blood in mainland Portugal. Participants, distributed proportionally by municipality and aged between 18 and 65 years, were selected randomly in 347 blood collection points between February and June 2022, and completed a sociodemographic and a KPP questionnaire. Detection of anti-*Leishmania* antibodies in serum was performed using an ELISA commercial kit. Individual KPP scores were calculated by adding grades defined for each question.

**Results:**

Globally, 201/3763 samples were positive. The estimated national true seroprevalence was 4.8% (95% CI 4.1–5.5%). The proportion of positive results was significantly different between NUTS (Nomenclature of Territorial Units for Statistics) regions. Models suggested that seropositivity was significantly higher in male sex, people older than 25 years, or residing in the Centro NUTS2 region, but not in dog owners nor people with lower KPP scores. Overall, 72.3% of participants had previously heard of leishmaniasis and, in multivariate analysis, a higher Knowledge score was associated with age 25–40 years, female sex, ownership of dogs, and higher education.

**Conclusions:**

Global estimated true seroprevalence (4.8%) was similar to previous regional studies in blood donors in neighboring Spain. Higher seroprevalence values in the NUTS2 Centro region were consistent with incidence data from humans and seroprevalence studies in dogs. On the other hand, the low values in the Alentejo and the high values in the northern subregions may be the result of geographical shifts in parasite circulation due to climate change and should prompt localized and integrated, vector, canine, and human research, following a One Health approach.

**Graphical Abstract:**

**Figure:**
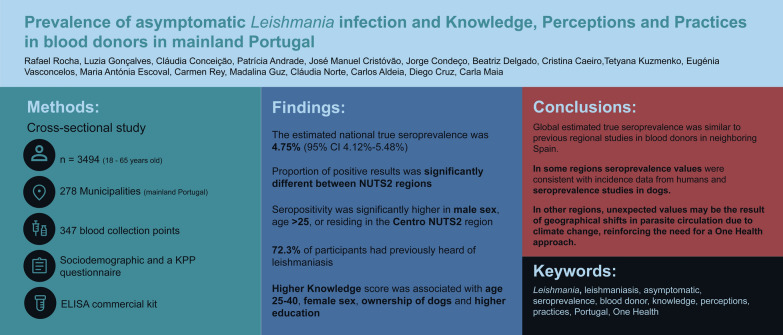

**Supplementary Information:**

The online version contains supplementary material available at 10.1186/s13071-023-05980-1.

## Background

Leishmaniases are a group of diseases caused by protozoa of the *Leishmania* genus, transmitted by phlebotomine sand fly bites. In the Mediterranean region, the parasites are maintained in a zoonotic cycle where dogs are the most important domestic reservoirs. In this context, the endemic species causing most cases of visceral leishmaniasis (VL) is *Leishmania infantum* [1, 2]. Systematic passive surveillance of VL cases in Portugal showed 6 to 14 cases per year between 2014 and 2018 [3], although this likely represents significant underreporting, as revealed in a previous study where only 38.6% of cases diagnosed in hospitals were notified to central public health authorities [4].

Infection by species of the *Leishmania donovani* complex, including *L. infantum*, is mostly recognized by its symptomatic presentations, of which VL is the most commonly described, although cutaneous and mucosal involvement are increasingly reported, especially in immunosuppressed individuals [5, 6]. However, many studies in *L. donovani* complex endemic areas suggest asymptomatic infection is the most common outcome of exposure to the parasite [7–9]. As for viscerotropic species, no single definition of asymptomatic *Leishmania* infection is universally accepted, different criteria have been used in healthy people (i.e., without signs/symptoms compatible with VL), namely detection of antibodies against *Leishmania*, detection of *Leishmania* DNA in blood, or a reactive leishmanin skin test or soluble *Leishmania* antigen test [10].

A systematic review included epidemiologic studies to identify and characterize asymptomatic infected individuals, as an indicator of areas and social contexts where active circulation and exposure to the parasite occur [11]. In the Mediterranean region, where *L. infantum* is endemic, several of these studies have been conducted not only in humans but also in animals, especially dogs. Serological techniques were often used to identify asymptomatic infection, and, in humans, target populations have frequently been blood donors, due to ease of access to samples and a higher confidence of the asymptomatic status, since blood donation is usually only permitted after strict symptom and behavior triage by health professionals and exclusion of anemia. In Spain, seroprevalence studies among the general population and blood donors have shown values between 1 and 8%, depending on the region [9, 12]. Factors associated with higher asymptomatic prevalence included older age, male sex, dog tutorship, contact with livestock (either at home or in the environment), living in rural communities, and living in a detached house [9, 13].

In Portugal, asymptomatic *Leishmania* infection in humans was only addressed in two studies, using serological testing, at a regional level and with a limited sample size. One of them found no seropositive individuals among 229 blood donors living in the “Beiras e Serra da Estrela” (BSE) NUTS3 (Nomenclature of Territorial Units for Statistics, from the French *Nomenclature des Unités territoriales statistiques*) region [14] while in the other only 2/100 healthy dog owners were seropositive in the “Área Metropolitana de Lisboa” (AML) [15]. Contrastingly, the geographical patterns of *Leishmania* infection in dogs in Portugal have been more extensively investigated. A recent nationwide study, involving 1860 dogs, showed a national estimated true seroprevalence of 12.5%, varying between 0 and 30.5% among districts [16] and revealed higher seroprevalence in some areas partially matching the known classical distribution of most human (and animal) symptomatic cases, including the Douro, Coimbra and Médio Tejo NUTS3 regions.

In spite of the wide distribution and significant burden on endemic populations of VL, knowledge, perceptions, and practices (KPP) regarding this clinical form are not homogeneous among countries and even different regions of the same country or different sectors of the population [17–19]. However, few studies have sought associations between KPP and asymptomatic infection. In Portugal, KPP studies have been directed to animal owners and were not coupled with seroprevalence analysis [20, 21]. In these studies, 83–91% of the owners had heard of animal leishmaniasis, but only 38.6% of human leishmaniasis. Hearing of leishmaniasis was significantly associated with non-rural areas and academic degree.

Therefore, this study aimed to estimate the national prevalence of asymptomatic *Leishmania* infection in blood donors and search for associations between the presence of anti-*Leishmania* antibodies and several sociodemographic factors in this population, as well as with the KPP of blood donors regarding leishmaniasis.

## Population, materials, and methods

### Study population and sample size calculation

This cross-sectional national study was carried out between February and June 2022 in blood donors in mainland Portugal, which is in Southwest Europe, bordering Spain and the Atlantic Ocean. According to the 2021 national census, the population of mainland Portugal aged 15 to 64 years was 6,257,752 inhabitants [22]. Mainland Portugal is divided into five NUTS2 regions, 23 NUTS3 regions (Additional file 1: Table S1), 278 municipalities (“municípios”), and 2882 parishes (“freguesias”). To ensure a nationwide coverage of sampling, this study was performed in collaboration with the Portuguese Institute of Blood and Transplantation (IPST) and with the immunohemotherapy departments (IHDs) of five hospital centers in the Alentejo and Algarve regions. The IPST and the IHDs perform regular blood collections in fixed centers as well as in shifting stations in rural and urban areas. In 2021, over 190,000 blood donations were performed in these institutions. Individuals are considered eligible for donation after a strict triage conducted by a trained health professional, to exclude acute disease and several chronic conditions and risk behaviors. Additionally, capillary hemoglobin levels are determined; men with less than 13.5 g/dl and women with less than 12.5 g/dl are automatically excluded from donating.

Sample size was estimated using the Epitools^©^ Epidemiological Calculators [23, 24]. At least 3200 individuals were needed to estimate a 95% confidence interval (CI) for prevalence, considering an expected maximum global (national) seroprevalence of 9% (based on Spanish regional studies [9, 13, 25]) and a minimum sensitivity and specificity of the serological test used of 85% and 90%, respectively, and considering a desired precision of 0.02 to a 95% CI. Additionally, this sample size would allow the detection of small differences in seroprevalence between NUTS3 regions, with a power of 95%, using a Chi-square test.

Sampling was stratified by municipality: the number of participants enrolled from each municipality was proportional to the fraction of the mainland population (aged 15–64 years) living in that region, according to the most recent census data and assuming a similar distribution for blood donors. For five NUTS3 regions in southern Portugal (Alto Alentejo, Alentejo Central, Baixo Alentejo, Alentejo Litoral, and Algarve), where higher seroprevalence was expected a priori (according to human incidence data derived from VL cases reported to the National Surveillance System [3]), recruitment of additional participants was planned in order to increase the precision of estimates, increasing the total sample size to 3494.

### Eligibility criteria

Individuals enrolled in this study presented to one of the institutions collaborating in the study from February to June 2022 and were considered fit for blood donation. Only individuals aged 18 to 65 years were included. Blood donation must have been completed, including collection of a serum sample for routine serological testing (for the following blood-borne pathogens: hepatitis B virus, hepatitis C virus, human immunodeficiency virus [HIV], *Treponema pallidum*).

### Data and sample collection

Participant enrollment was performed in non-randomly selected blood collection sessions, to ensure a maximum number of municipalities were surveyed. In some municipalities, more than one session was required to complete the calculated sample sizes, and, in this case, different zones of the municipality were preferably surveyed. Blood donation sessions exclusive to specific professional groups (such as police officers or firefighters) were generally avoided. A fixed number of participants was set for each session (1 to 8). Blood donors were randomly invited to the study, according to hour of presentation at the blood collection center/station–considering non-consecutive, pre-defined time slots. This procedure differed in one center (in the Lisbon Metropolitan Area), due to logistic reasons, where all donors in each session were invited consecutively, by order of arrival, until the sample size for the municipality was fulfilled.

Recruitment was performed in both fixed donation centers and mobile stations, except in the Algarve region. The participants were informed about the study and signed an informed consent declaration. Each participant completed a self-administered structured paper questionnaire about sociodemographic aspects and KPP regarding leishmaniasis (Additional file 2: Fig. S1). This questionnaire was pretested in a convenience sample of 40 blood donors from the Norte and AML regions. 1.5 ml of serum taken from the peripheral blood sample collected for routine serological testing was sent to the Instituto de Higiene e Medicina Tropical (IHMT) and stored at − 20 ºC for the study.

Categorical variables extracted from the questionnaire were analyzed mostly using the original categories provided as answer options, but regrouping was performed in some cases. Classification of professions was performed using the European Skills, Competences, and Occupations (ESCO) classification of occupations, developed by the European Commission in 2010 [26]. NUTS regions, municipalities, parishes, and unions of parishes were defined according to the most recent organizational definition, published in 2013 and implemented in 2015. The order of presentation of NUTS2 and 3 regions in tables follows the numerical and/or alphabetical order of their respective official codes. Classification of parishes as rural or non-rural followed the Portuguese Rural Development Program 2014–2020 [27]. *Leishmania donovani* complex-endemic travel destinations included countries in Europe where disease has been reported in most or all regions: Albania, Bulgaria, Cyprus, Greece, Italy, Malta, and Spain [2]. And outside Europe, countries where over 200 cases of VL were reported to the World Health Organization (WHO) in 2021 [28] include Brazil, Eritrea, Ethiopia, India, Kenya, Nepal, Somalia, South Sudan, Sudan, and Yemen.

### Serological study

Antileishmanial antibody detection in each serum sample was performed using enzyme-linked immunosorbent assay (ELISA) (*Leishmania* ELISA IgG+IgM, Vircell^®^, Spain), following the manufacturer’s instructions and cut-offs. These kits simultaneously detect immunoglobulin M (IgM) and/or IgG antibodies against *Leishmania*; the wells of the plate are coated with an unspecified *L. infantum* antigen. The sensitivity and specificity of the ELISA, according to the manufacturer, are 97% and 99%, respectively. A single determination was performed for each serum sample. Samples were classified as positive, negative, or borderline (when optical density was less than 10% lower or higher than the average value of the borderline controls). Participants from whom positive samples were taken were considered to have been exposed to *Leishmania*—either past or current asymptomatic infection. Positive or borderline results were tested by a second method (in-house immunofluorescence antibody test [IFAT]) and, if these samples showed a titer of 1:64 or higher, participants were informed of the result (if they had expressed this wish in the consent declaration).

### Statistical analysis

True prevalence was estimated at the regional level considering only positive samples and based on the following formula: true prevalence (TP) = (test prevalence – 1 + specificity)/(sensitivity – 1 + specificity), considering the values provided by the manufacturer. The corresponding 95% CIs were obtained using Wilson’s method on Epitools^©^ Epidemiological Calculators [23, 29]. For finer detail geographical analysis (at municipality or parish level), however, borderline samples were also considered, since it was assumed they could represent some degree of exposure to the parasite.

Absolute and relative frequencies and hypothesis testing were performed using IBM^®^ SPSS^®^ Statistics Version 29.0. Bar charts were built using Microsoft^®^ Excel^®^. Geographical representation and analysis of results were obtained using QGIS^®^ Version 3.22. Answers to each KPP question were scored according to the criteria presented in Additional file 3: Table S2. A total score was calculated for knowledge (*K* score), perceptions (Per score), and practices (Pra score), by adding the individual scores of all the questions in each category. The range of possible values for each score was as follows: *K* 0–19; Per 0–6; Pra 0–6.

Descriptive statistics were expressed as absolute frequencies and percentages for categorical variables and as means with standard deviations or medians with interquartile ranges (IQRs) for continuous variables (e.g., age, *K*, Per, and Pra scores). Comparisons between groups were performed using the Pearson Chi-square test for categorical variables (or Fisher’s exact in case of failure of the assumptions of the *χ*^2^ test). For continuous variables, after checking the assumptions of normality and homogeneity of the variances, instead of *t*-test and analysis of variance (ANOVA), the Mann–Whitney *U* test or the Kruskal–Wallis test were used, for comparing two or more independent groups, respectively. A value of *P* < 0.05 was considered statistically significant.

Multivariate analyses were conducted to identify sociodemographic factors and KPP associated with asymptomatic infection, and sociodemographic factors associated with higher *K*, Per, or Pra scores. These analyses were performed through multiple binary logistic regression models, analyzing variables with statistical meaning in the univariate analysis (*P* < 0.05) and some biologically relevant or potentially confounding variables.

For those variables that remained significant, the crude odds ratio (OR) was updated to adjusted odds ratio (aOR) with 95% CI. The Hosmer–Lemeshow test was used for assessing goodness of fit in each multiple logistic regression model [30]. To determine potential risk factors for positive ELISA results, only two groups were considered: positive and negative samples (borderline samples were excluded). To determine factors associated with higher *K*, Per, or Pra scores, donors were divided into two groups for each score: above the global median score value, and equal to or below this value (*K* = 7, Per = 0, Pra = 3.5). The reference categories used for each independent variable are specified in each multivariate analysis results table.

## Results

**Fig. 1:**
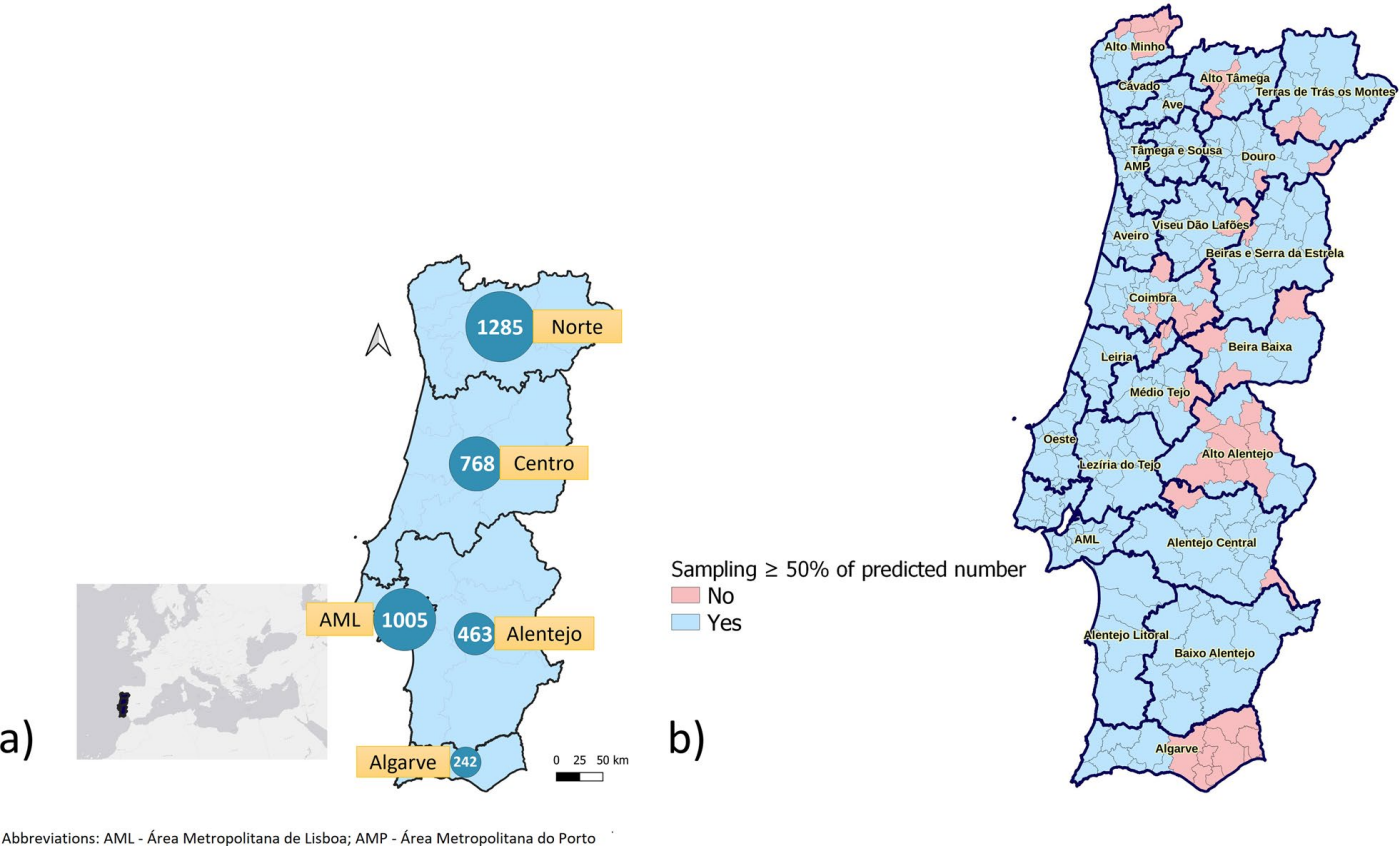
**a** Number of participants recruited by NUTS2 region (circle sizes are proportional to the number of participants recruited); **b** municipalities where at least 50% of the calculated sample size was achieved

In total, 3763 participants were included in this study. Participants were recruited in 636 blood collection sessions, in 347 different collection sites. The number of participants recruited by NUTS2 region is presented in Fig. 1a, and the municipalities where at least 50% of the calculated sample size was achieved are displayed in Fig. 1b. Missing municipalities were concentrated in the eastern Algarve, Alto Alentejo, Coimbra, and Alto Minho NUTS3 regions.

### Sociodemographic characteristics

The median age was 41 years, with significant differences between NUTS2 regions (older in the Alentejo and Algarve) (Table 1; more detailed information by NUTS3 region is presented in the Additional file 4: Table S3). Participants were evenly distributed between sexes globally and in most regions, except for the Alentejo and the Algarve, where male sex was clearly predominant. Education level was significantly higher in the AML (Chi-square test, *χ*^2^ = 122.2, *df* [degrees of freedom] = 16, *P* < 0.001). Globally, 23.7% of participants mentioned traveling or living abroad in the previous 2 years, mostly (58.6%) to at least one of the *L. donovani* complex endemic countries listed in the “Materials and methods” section.

**Table 1:** Sociodemographic characteristics of the participants, globally and by NUTS (Nomenclature of Territorial Units for Statistics) 2 region

	Global	Norte	Centro	AML	Alentejo	Algarve	*P*-value
Total	100	34.1	20.4	26.7	12.3	6.4	
	(3763/3763)	(1285/3763)	(768/3763)	(1005/3763)	(463/3763)	(242/3763)	
Median age (*years*)	41	39	41	42	43	43	< 0.001* (*H* = 40.7, *df* = 4)
(IQR)	(31–48)	(30–47)	(29–48)	(31–50)	(34–50)	(35–50)	
Male sex (%)	49.8	47.6	48.2	47.7	59.4	57.1	< 0.001* (*χ*^2^ = 27.3, *df* = 4)
	(1867/3749)	(609/1280)	(369/765)	(478/1003)	(274/461)	(137/240)	
Education level^a^							
Basic (1–4)	1.9	1.6	2.8	1.2	2.7	1.7	< 0.001* (*χ*^2^ = 122.2, *df* = 16)
	(69/3659)	(20/1240)	(21/749)	(12/983)	(12/451)	(4/236)	
Basic (5–9)	16.8	20.7	16.3	10.8	20.0	16.9	
	(615/3659)	(257/1240)	(122/749)	(106/983)	(90/451)	(40/236)	
Secondary (10–12)	44.1	43.5	43.9	39.4	51.9	51.7	
	(1612/3659)	(540/1240)	(329/749)	(387/983)	(234/451)	(122/236)	
Bachelor's	25.8	22.9	27.6	32.3	19.3	20.3	
	(944/3659)	(284/1240)	(207/749)	(318/983)	(87/451)	(48/236)	
MSc/PhD	11.5	11.2	9.3	16.3	6.2	9.3	
	(419/3659)	(139/1240)	(70/749)	(160/983)	(28/451)	(22/236)	
Occupation							
Student	9.9	8.9	13.3	11.0	6.9	4.2	
	(295/2993)	(90/1015)	(84/630)	(88/797)	(25/361)	(8/190)	
Unemployed	3.4	4.3	3.3	3.3	1.1	3.2	
	(101/2993)	(44/1015)	(21/630)	(26/797)	(4/361)	(6/190)	
Employed^b^	84.9	85.4	80.6	83.3	88.9	90.0	
	(2541/2993)	(867/1015)	(508/630)	(664/797)	(321/361)	(171/190)	
Armed forces (0)	2.2	1.5	2.8	2.1	2.8	4.1	< 0.001* (*χ*^2^ = 128.9, *df* = 12)
	(57/2541)	(13/867)	(14/508)	(14/664)	(9/321)	(7/171)	
Managers, professionals, and technicians (1–3)	46.0	42.7	43.1	59.8	35.5	34.5	
	(1169/2541)	(370/867)	(219/508)	(397/664)	(114/321)	(59/171)	
Clerical support, service, and sales (4–5)	30.4	29.3	28.3	25.0	38.6	49.7	
	(773/2541)	(254/867)	(144/508)	(166/664)	(124/321)	(85/171)	
Agriculture, craft, industry, and elementary (6–9)	21.3	26.5	25.8	13.1	23.1	11.7	
	(542/2541)	(230/867)	(131/508)	(87/664)	(74/321)	(20/171)	
Travel abroad							
Yes (previous 2 years)	23.7	24.9	22.1	26.8	17.5	21.3	0.001* (*χ*^2^ = 17.9, *df* = 4)
	(874/3689)	(310/1247)	(167/755)	(267/996)	(79/452)	(51/239)	
≥ 1 Endemic country ^c^	58.6	52.3	54.4	54.7	76.0	55.3	0.015* (*χ*^2^ = 12.4, *df* = 4)
	(450/768)	(162/310)	(81/149)	(129/236)	(57/75)	(21/38)	

*AML* Área Metropolitana de Lisboa, *IQR* interquartile range, *MSc* Master of Science, *PhD* Doctor of Philosophy

*Statistically significant

^a^ Numbers in brackets refer to number of years completed of formal school education

^b^ Numbers in brackets refer to the numbers of the categories in the classification of European Skills, Competences, and Occupations

^c^ Albania, Brazil, Bulgaria, Cyprus, Eritrea, Ethiopia, Greece, India, Italy, Kenya, Malta, Nepal, Somalia, South Sudan, Spain, Sudan, Yemen

^d^ According to the results of the 2021 National Census

### Serological results

In total, 201 (5.3%) samples were positive by ELISA and 97 (2.6%) were borderline. The distribution of positive results by NUTS2 and 3 regions is represented in Table 2. Adjusted positivity rates considered slight deviations between the expected and the achieved sample size by municipality.

**Table 2:** Distribution of positive results by NUTS (Nomenclature of Territorial Units for Statistics) 2 and 3 region and estimated true prevalence

Region	Sampling sites (*n*)	Samples (*n*)	Positive samples (*n*)	Crude positivity rate (%)	Adjusted positivity rate (%)	True prevalence (%)	95% Confidence interval
Norte	149	1285	82	6.4	6.3	5.5	4.4–6.9
Alto Minho	12	72	6	8.3	8.5	7.8	3.6–16.0
Cávado	17	160	6	3.8	3.5	2.6	1.0–6.6
Ave	16	155	9	5.8	5.4	4.6	2.3–9.3
Área Metropolitana do Porto	60	591	44	7.4	7.4	6.6	4.9–8.9
Alto Tâmega	4	29	0	0.0	0.0	0.0	0–11.7
Tâmega e Sousa	23	163	9	5.5	5.6	4.8	2.5–9.3
Douro	13	79	6	7.6	7.9	7.2	3.4–14.9
Terras de Trás-os-Montes	4	36	2	5.6	2.0	1.1	0.2–6.0
Centro	119	768	51	6.6	6.5	5.8	4.3–7.6
Oeste	12	145	8	5.5	4.9	4.1	1.9–8.6
Região de Aveiro	19	146	12	8.2	7.7	6.9	3.8–12.3
Região de Coimbra	21	114	7	6.1	6.1	5.3	2.5–11.1
Região de Leiria	14	103	6	5.8	6.2	5.4	2.5–11.4
Viseu Dão-Lafões	15	67	3	4.5	4.2	3.3	0.9–11.4
Beira Baixa	7	33	2	6.1	5.9	5.1	1.4–16.9
Médio Tejo	16	73	6	8.2	9.8	9.2	4.5–17.8
Beiras e Serra da Estrela	15	87	7	8.0	8.8	8.1	4.0–15.7
Área Metropolitana de Lisboa	25	1005	47	4.7	4.9	4.0	3.0–5.4
Alentejo	52	463	9	1.9	1.9	0.9	0.4–2.3
Alentejo Litoral	10	70	1	1.4	1.3	0.3	0.0–5.2
Baixo Alentejo	8	105	1	1.0	0.8	0.0	0.0–3.5
Lezíria do Tejo	10	87	2	2.3	2.3	1.4	0.3–7.5
Alto Alentejo	9	57	1	1.8	1.4	0.5	0.0–6.3
Alentejo Central	15	144	4	2.8	2.7	1.8	0.6–5.1
Algarve	2	242	12	5.0	4.2	3.3	1.7–6.4
Total	347	3763	201	5.3	5.6	4.8	4.1–5.5

**Fig. 2:**
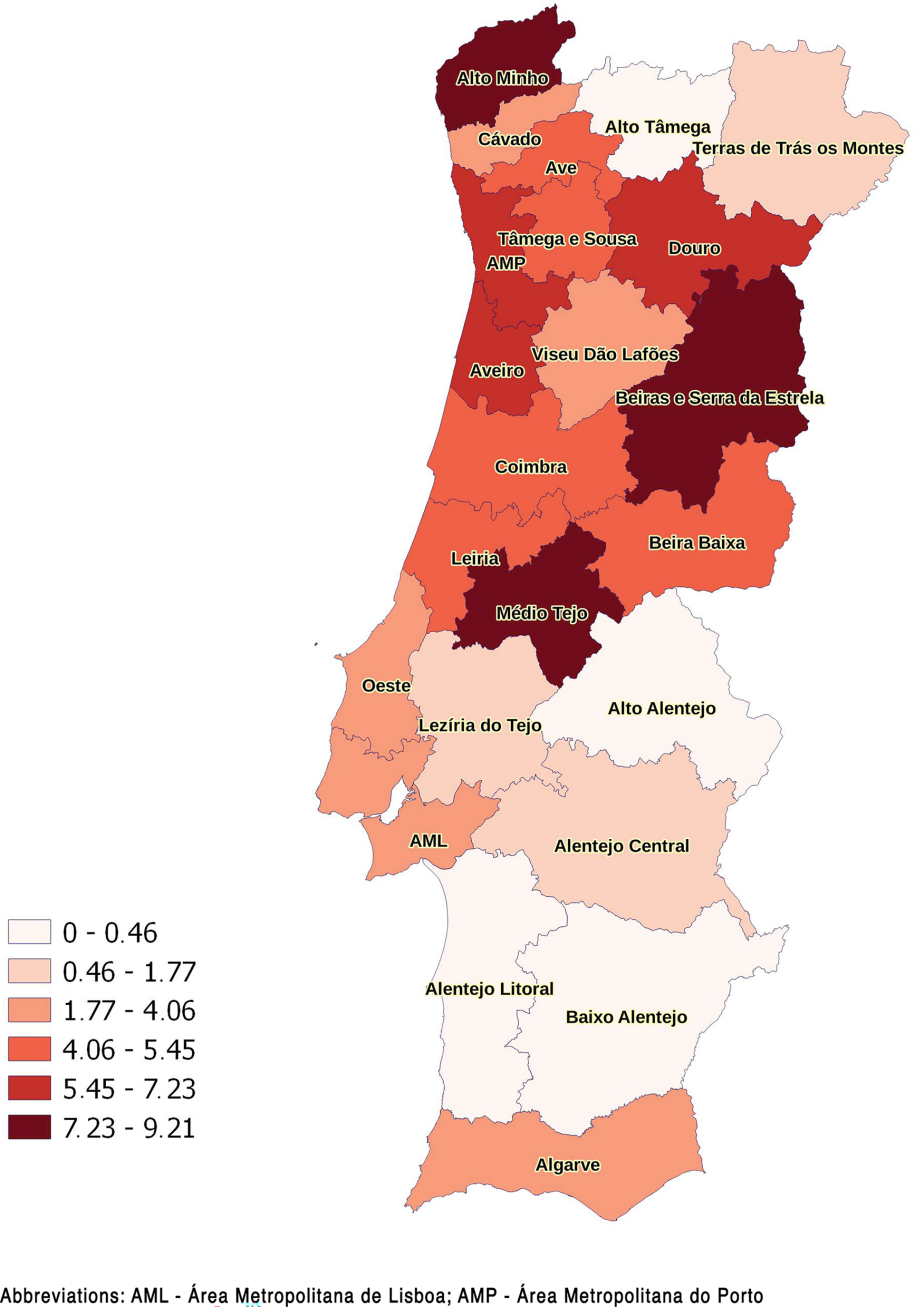
Distribution of estimated true seroprevalence values (%) by NUTS3 region

Figure 2 shows the distribution of true seroprevalence estimates by NUTS3 region. Global estimated true seroprevalence was 4.8%. At the NUTS3 level, values ranged from 0.0 to 9.2%, with the highest seroprevalence in the Médio Tejo, Alto Minho, and BSE regions. There was a statistically significant difference in the proportion of positive results between NUTS2 regions (Chi-square test, *χ*^2^ = 17.7, *df* = 4, *P* = 0.001), but this difference was not significant between NUTS3 regions within the Norte, Centro, and Alentejo NUTS2 regions (*P*-values of 0.519, 0.949, and 0.896, respectively).

**Fig. 3:**
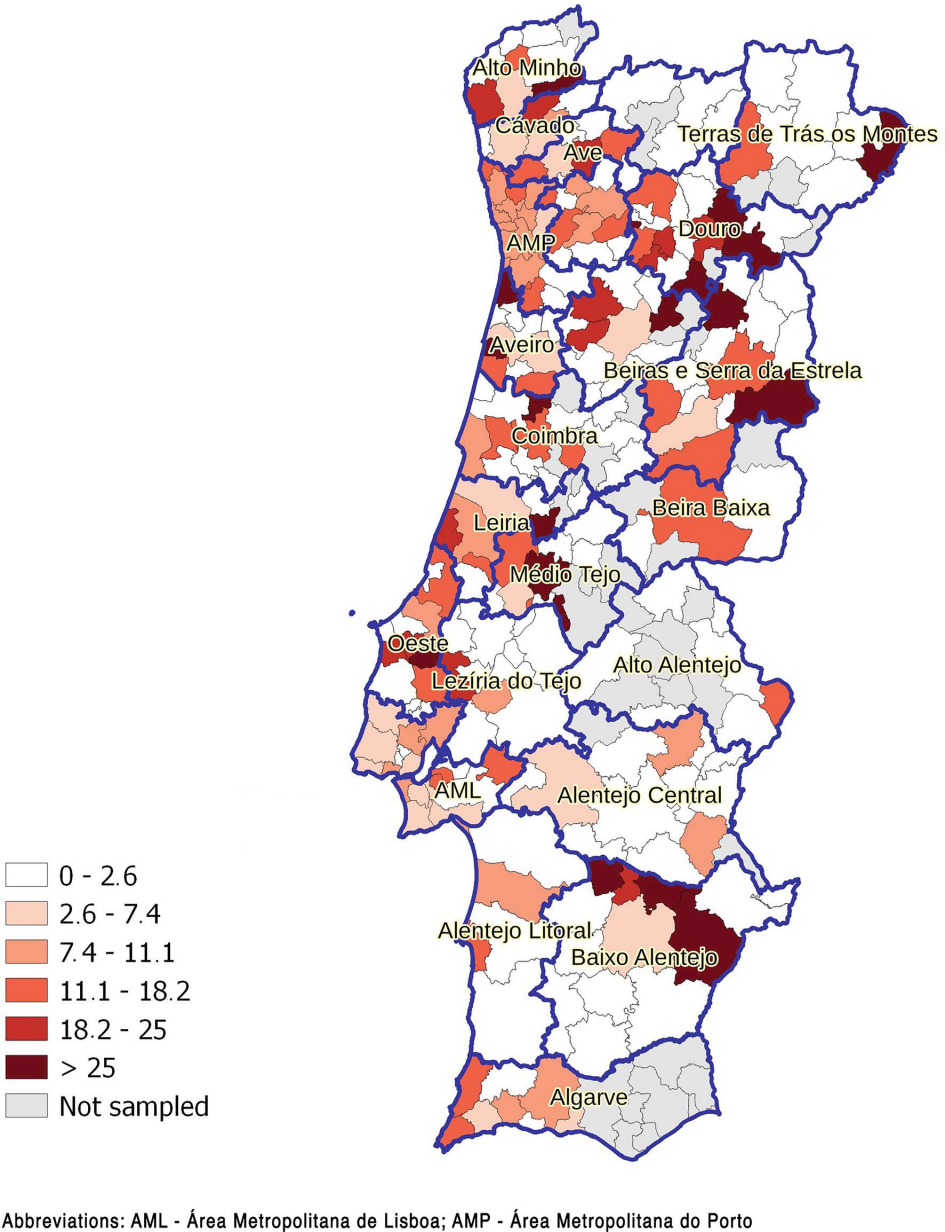
Percentage of positive or borderline samples by municipality (natural breaks were used to define categories)

To allow a more detailed analysis of the geographical distribution of possible exposure to *Leishmania*, the percentage of positive or borderline samples was calculated by municipality and is presented in Fig. 3. Municipalities where over 20 samples were collected (*n* = 53) with the highest percentages (> 13%) were (by descending order) Ílhavo, Viana do Castelo, Oliveira de Azeméis, Penafiel, Alcobaça, Alenquer, Moita, Póvoa de Varzim, Vila Real, and Paços de Ferreira.

**Fig. 4:**
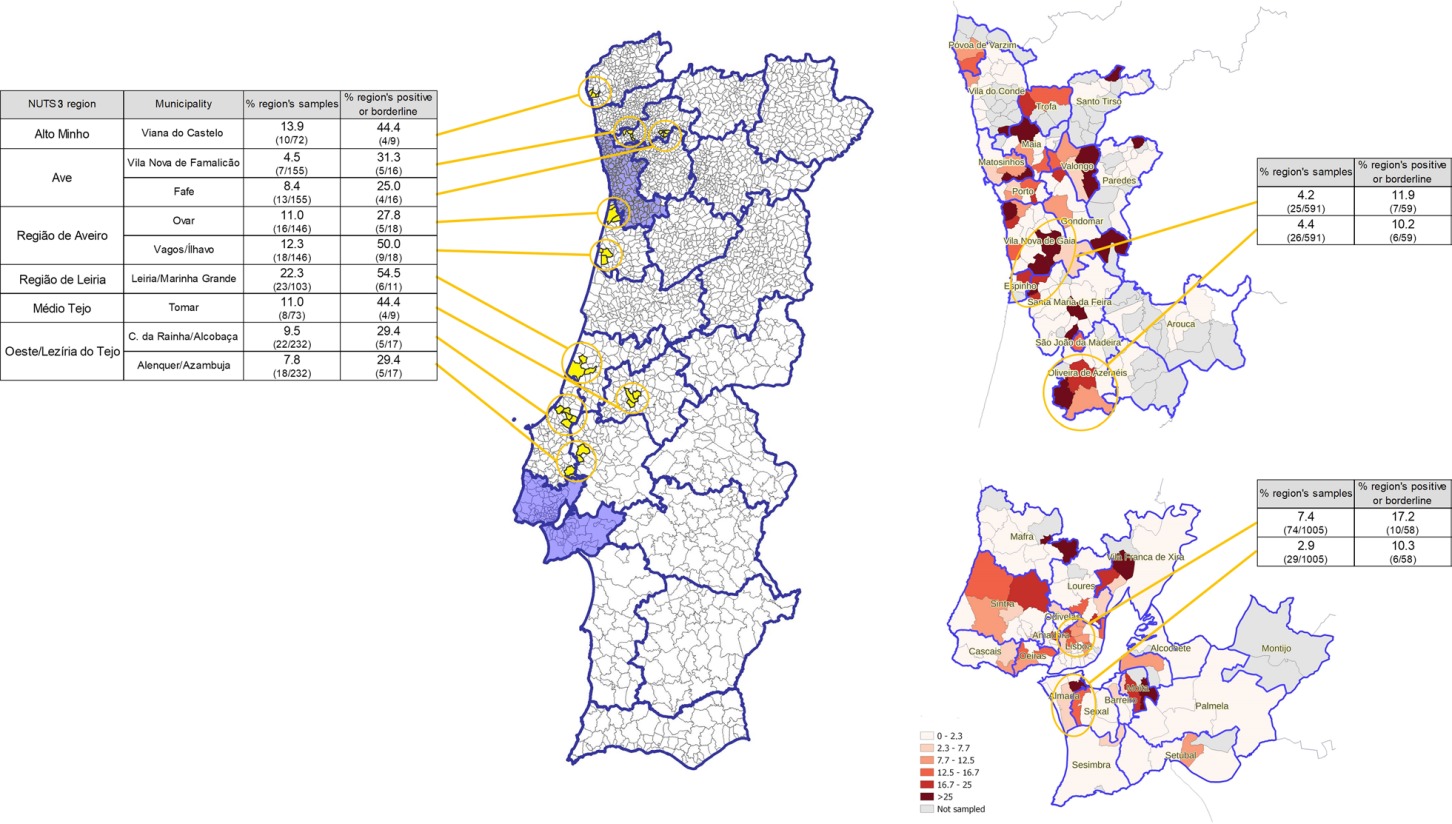
Percentage of positive or borderline samples, in each parish of the “Área Metropolitana de Lisboa” and “Área Metropolitana do Porto” regions (right) and in some selected groups of parishes in other NUTS3 regions

To understand possible clustering of positive/borderline cases at a more local level, percentage by parish is illustrated for the AML and “Área Metropolitana do Porto” (AMP) NUTS3 region in Fig. 4. For other NUTS3 regions, geographical coverage to such a finer detail was not possible; however, some groups of contiguous parishes represented a high percentage of the total number of positive/borderline samples within their respective NUTS3 region (represented in yellow in Fig. 4).

### Knowledge, perceptions, and practices

Answers to individual KPP questions are summarized in Table 3 (more detailed information by NUTS3 region is presented in the Additional file 5: Table S4).

**Table 3:** Answers to knowledge, perceptions, and practices questions, globally and by NUTS2 region

	Global (%)	Norte (%)	Centro (%)	AML (%)	Alentejo (%)	Algarve (%)	*P*-value
Total (*n*)	3763	1284	769	1006	462	242	
Had heard of leishmaniasis	72.3	61.9	78.1	77.5	78.1	75.9	< 0.001* (*χ*^2^ = 103.9, *df* = 4)
	(2704/3740)	(791/1277)	(596/763)	(777/1002)	(357/457)	(183/241)	
Source of information							
Television	53.2	61	52.3	51.2	49.7	38.1	
	(1406/2643)	(469/769)	(307/587)	(386/754)	(175/352)	(69/181)	
Veterinarian	48.1	39.5	48.9	52	52.8	57.5	
	(1273/2643)	(304/769)	(287/587)	(392/754)	(186/352)	(104/181)	
Cause							
Infectious	68.8	66.5	71.8	69.6	67.2	68.7	0.685 (*χ*^2^ = 5.1, *df* = 4)
	(1817/2640)	(517/777)	(416/579)	(530/761)	(231/344)	(123/179)	
Route of transmission							
DK/CR	19.5	21	18	19.3	20.7	15.8	
	(526/2704)	(166/791)	(107/596)	(150/777)	(74/357)	(29/183)	
Knows							
Arthropod bite	88.2	88.3	89.8	86.8	86.9	91.6	
	(1922/2178)	(552/625)	(439/489)	(544/627)	(246/283)	(141/154)	
Sand fly bite	13.5	12.7	13.7	15.9	11.8	6.4	
	(260/1922)	(70/552)	(60/439)	(92/544)	(29/246)	(9/141)	
Contact with animals	19.6	20.6	19.4	20.1	19.4	13.6	
	(426/2178)	(129/625)	(95/489)	(126/627)	(55/283)	(21/154)	
Affects animals	91	86.3	93.6	93.2	93	89.6	< 0.001* (*χ*^2^ = 32.4, *df* = 4)
	(2451/2693)	(676/783)	(557/595)	(722/775)	(332/357)	(164/183)	
Clinical signs in animals							
DK/CR	46.5	49.4	44.2	48.8	43.4	38.4	
	(1139/2451)	(334/676)	(246/557)	(352/722)	(144/332)	(63/164)	
Knows							
Weight loss	52.3	43.9	49.8	56.2	64.9	50.5	
	(686/1312)	(150/342)	(155/311)	(208/370)	(122/188)	(51/101)	
Skin lesions	48.2	51.5	45.3	51.6	40.4	48.5	
	(633/1312)	(176/342)	(141/311)	(191/370)	(76/188)	(49/101)	
Treatable in animals	62.8	63.7	63.3	59	64.6	70.3	0.032* (*χ*^2^ = 10.4, *df* = 4)
	(1397/2226)	(393/617)	(317/501)	(393/666)	(192/297)	(102/145)	
Lethal in animals	72.1	62.3	74.5	74.4	75.4	84.3	< 0.001* (*χ*^2^ = 40.1, *df* = 4)
	(1487/2063)	(336/539)	(350/470)	(472/634)	(215/285)	(113/134)	
Preventable in animals	78	74.8	77.9	78.1	80.1	86	0.057 (*χ*^2^ = 9.2, *df* = 4)
	(1605/2059)	(415/555)	(363/466)	(489/626)	(221/276)	(117/136)	
Present in Portugal	86.1	82.2	86.2	87.8	88.1	90.2	0.010* (*χ*^2^ = 13.8, *df* = 4)
	(2081/2418)	(544/662)	(473/549)	(628/715)	(289/328)	(148/164)	
Species affected							
DK/CR	6.9	8.1	5.5	7.6	5.5	6.8	
	(144/2081)	(44/543)	(26/473)	(48/628)	(16/289)	(10/148)	
Knows							
Dogs	97.4	97.6	96.6	97.6	96.7	100	
	(1893/1937)	(487/499)	(432/447)	(566/580)	(264/273)	(138/138)	
Cats	32.6	43.1	33.3	28.8	26	21.7	
	(632/1937)	(215/499)	(149/447)	(167/580)	(71/273)	(30/138)	
Affected animals close to household	11.6	6.4	12.2	14	13.5	15.8	< 0.001* (*χ*^2^ = 22.4, *df* = 4)
	(240/2069)	(34/534)	(57/468)	(88/630)	(38/281)	(23/146)	
Vaccine available	52.2	43.7	53.6	54.6	56.3	62	< 0.001* (*χ*^2^ = 25.3, *df* = 4)
	(1092/2090)	(238/545)	(253/472)	(347/636)	(161/286)	(93/150)	
Possible importation	61.6	58.9	61.5	64.6	58.5	65.2	0.174 (*χ*^2^ = 7.0, *df* = 4)
	(1477/2399)	(387/657)	(334/543)	(460/712)	(189/323)	(107/164)	
Affects humans	53.8	56.4	51.9	51.6	56.7	51.6	0.333 (*χ*^2^ = 6.0, *df* = 4)
	(1433/2666)	(438/776)	(305/588)	(396/767)	(200/353)	(94/182)	
Organs affected							
DK/CR	57.1	55.7	56.4	60.1	58	51.1	
	(818/1433)	(244/438)	(172/305)	(238/396)	(116/200)	(48/94)	
Knows							
Skin	61.8	69.6	63.9	62	47.6	50	
	(380/615)	(135/194)	(85/133)	(98/158)	(40/84)	(23/46)	
Liver/spleen	37.7	30.9	33.1	48.7	46.4	28.3	
	(232/615)	(60/194)	(44/133)	(77/158)	(39/84)	(13/46)	
Treatable in humans	55.6	59.6	56.8	52.2	53.2	53.3	0.652 (*χ*^2^ = 5.4, *df* = 4)
	(772/1388)	(251/421)	(168/296)	(206/395)	(99/186)	(48/90)	
Lethal in humans	35.4	32.3	38.2	32.9	40.6	41	0.295 (*χ*^2^ = 6.5, *df* = 4)
	(438/1236)	(118/365)	(99/259)	(118/359)	(69/170)	(34/83)	
Preventable in humans	56.2	58.2	58.1	53	55.5	57.3	0.222 (*χ*^2^ = 6.7, *df* = 4)
	(705/1254)	(217/373)	(151/260)	(194/366)	(96/173)	(47/82)	
Present in Portugal	78.7	77.6	84.6	74.5	78.7	83	0.042* (*χ*^2^ = 12.1, *df* = 4)
	(1135/1442)	(339/437)	(259/306)	(304/408)	(155/197)	(78/94)	
Possible importation	70.2	72.1	72.9	68.2	66.8	68.1	0.450 (*χ*^2^ = 103.9, *df* = 4)
	(1005/1432)	(313/434)	(223/306)	(274/402)	(131/196)	(64/94)	
Regular contact with wild animals	4.3	2.6	5.9	3	8.1	5.6	< 0.001* (*χ*^2^ = 32.1, *df* = 4)
	(144/3387)	(30/1156)	(40/673)	(28/925)	(34/419)	(12/214)	
Regular contact with domestic animals	70.5	70	77.7	63.6	74.1	73.6	< 0.001* (*χ*^2^ = 44.7, *df* = 4)
	(2524/3578)	(848/1212)	(565/727)	(616/969)	(326/440)	(170/231)	
Regular nighttime outdoor activities	24.2	19.7	28.4	23.1	30.4	27.8	< 0.001* (*χ*^2^ = 30.2, *df* = 4)
	(825/3409)	(229/1162)	(195/686)	(214/928)	(127/418)	(60/216)	
Nets in some/all windows/doors	23.7	12.9	25.2	23	45	38.6	< 0.001* (*χ*^2^ = 217.0, *df* = 4)
	(848/3572)	(157/1213)	(184/730)	(221/962)	(198/440)	(88/228)	
Dog ownership	48.1	46.3	57.6	39.5	55.4	50.2	< 0.001* (*χ*^2^ = 67.6, *df* = 4)
	(1775/3688)	(578/1248)	(433/752)	(394/997)	(251/453)	(119/237)	
Dog outdoors during nighttime	63.8	60.5	69.2	63.7	62.9	62	0.082 (*χ*^2^ = 8.3, *df* = 4)
	(1055/1653)	(321/531)	(277/400)	(239/375)	(151/240)	(67/108)	
Use of repellents/insecticides	82.2	80.7	83.2	86.2	80.9	76.2	0.096 (*χ*^2^ = 7.9, *df* = 4)
	(1320/1605)	(413/512)	(326/392)	(312/362)	(190/235)	(80/105)	
Spot-on	55	60	58	51	45.8	55	
	(726/1320)	(248/413)	(189/326)	(159/312)	(87/190)	(44/80)	
Collar	40.9	32	40.5	49	47.9	40	
	(540/1320)	(132/413)	(132/326)	(153/312)	(91/190)	(32/80)	
All year round	62.8	63.2	59.5	66.7	58.9	67.5	0.090 (*χ*^2^ = 5.5, *df* = 4)
	(829/1320)	(261/413)	(194/326)	(208/312)	(112/190)	(54/80)	
Regular veterinary follow-up	90.6	89.3	87.9	95.1	88.9	95.3	0.002* (*χ*^2^ = 16.8, *df* = 4)
	(1472/1625)	(466/522)	(348/396)	(349/367)	(209/235)	(101/106)	
Use of vaccine against canine leishmaniasis every year	21.7	14.4	20.6	28.2	25.5	31.9	< 0.001* (*χ*^2^ = 37.9, *df* = 4)
	(385/1775)	(83/578)	(89/433)	(111/394)	(64/251)	(38/119)	

*AML* Área Metropolitana de Lisboa, *DK/CR* don't know/can't remember

*Statistically significant

**Fig. 5:**
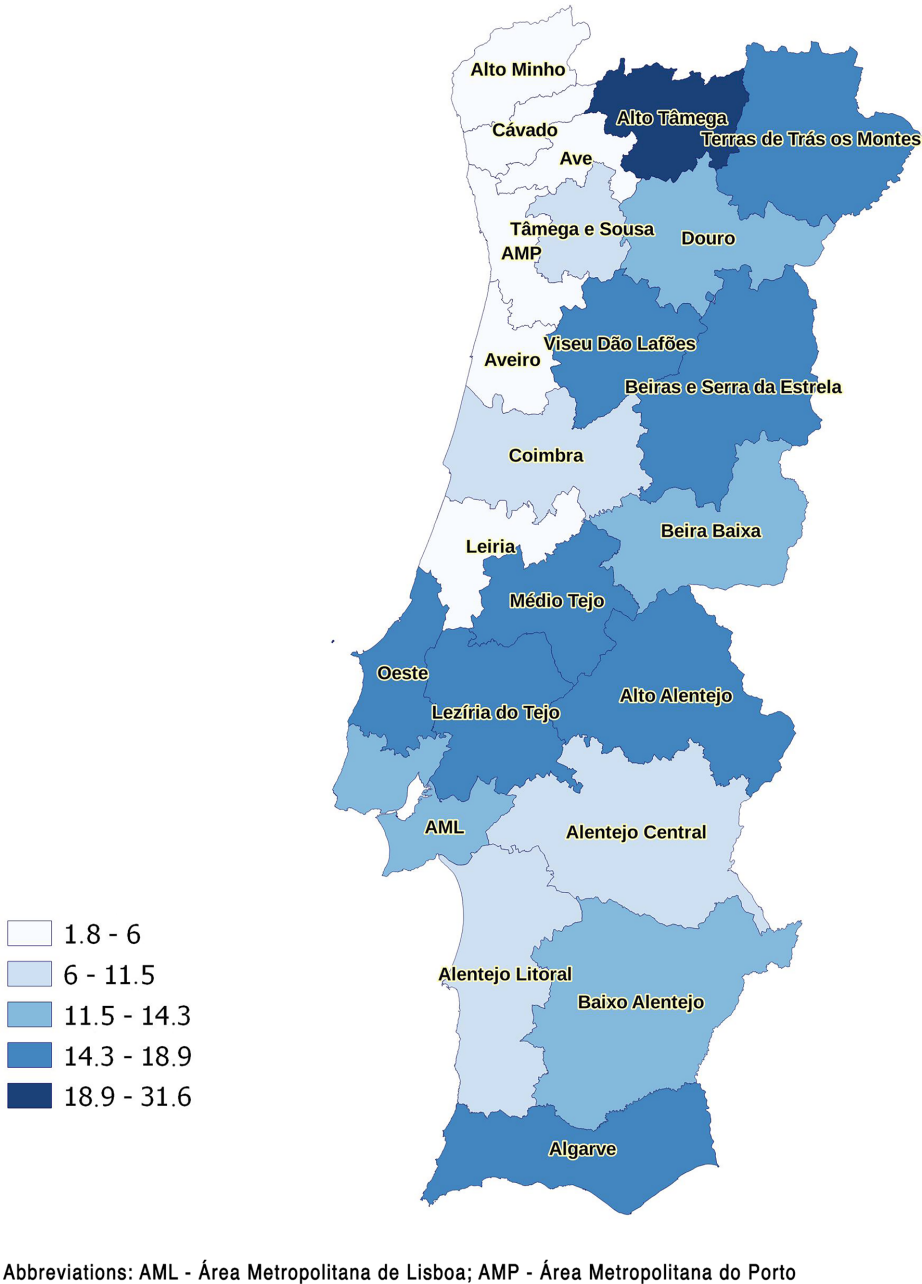
Percentage of donors reporting knowing animals with leishmaniasis in the household or nearby, by NUTS3 region

Globally, 72.3% of blood donors said they had previously heard of leishmaniasis, varying significantly among NUTS2 regions (Chi-square test, *χ*^2^ = 103.9, *df* = 4, *P* < 0.001) (lower in the Norte region, also with marked intra-regional variations: from 58.1% in the Ave to 81.0% in the Douro region). The most commonly reported sources of information about leishmaniasis were television (53.2%) and conversation with a veterinarian (48.1%). Television was reported as the predominant source in littoral NUTS3 regions of the Norte and Centro, whereas conversation with a veterinarian clearly dominated in the Algarve. Other sources are summarized in Additional file 7: Fig. S3a. Most people who admitted having previously heard of leishmaniasis (68.8%) recognized the infectious cause of the disease, and approximately 80% reported knowing the route of transmission: arthropod bite was most commonly selected, but direct contact with animals was simultaneously or alternatively selected in a significant proportion of cases (approximately 20%). Mosquitoes were the arthropods most commonly associated with transmission (only 13.5% of respondents selected sand flies). Other routes of transmission are summarized in Additional file 7: Fig. S3b. Understanding of routes of transmission was similar across different NUTS2 and NUTS3 regions. Most donors who admitted having previously heard of leishmaniasis (91.0%) recognized the disease affects animals, but only 53.8% admitted it could be a human disease. Only around 55%/45% of donors admitted knowing signs or symptoms of disease in animals/humans, respectively; in animals, weight loss (52.3%) and skin lesions (48.2%) were the signs most often selected (with skin lesions favored in the Norte and weight loss in the rest of the country); in humans, involvement of the skin was the most commonly recognized presentation of disease (61.8%). Other signs/symptoms of disease reported are represented in Additional file 7: Fig. S3c, d. Leishmaniasis was assumed as a lethal disease much more commonly for animals (72.1%) than for humans (35.4%), although knowledge of lethality in animals differed significantly among regions and was lower in the Norte (Chi-square test, *χ*^2^ = 40.1, *df* = 4, *P* < 0.001). The disease was generally reported as treatable and preventable both in animals and in humans, but more donors admitted it could be prevented in animals (78.0%) than in humans (56.2%). Only around 50% of donors recognized vaccination as a possible prevention strategy in animals; knowledge regarding vaccination was significantly higher in the Algarve and lower in the Norte region (Chi-square test, *χ*^2^ = 25.3, *df* = 4, *P* < 0.001). Disease was considered endemic in Portugal in animals (86.1%) or in humans (78.7%) by most donors who were aware these groups could be affected. In Portugal, dogs were almost universally pointed as the animals affected by leishmaniasis (97.4%), followed by cats (32.6%). Among participants who knew the disease was endemic in animals in Portugal, 11.6% assumed there were or had been diseased animals in the household or nearby. This percentage was significantly different between NUTS3 regions (Chi-square test, *χ*^2^ = 22.4, *df* = 4, *P* < 0.001), ranging from 1.8% (Ave) to 31.6% (Alto Tâmega), as shown in Fig. 5. Only four donors stated they knew people with leishmaniasis nearby: three in the AML and one in the Alto Alentejo.

Concerning practices, contact with domestic animals was commonly reported (70.5%) and almost half the donors were dog owners, being both significantly more common in the Centro and Alentejo regions. Regular follow-up of dogs (at least once a year) by a veterinarian was indicated by 90.6% of owners (higher in the Algarve and the AML, Chi-square test, *χ*^2^ = 16.8, *df* = 4, *P* = 0.002) but only 21.7% reported yearly vaccination of dogs against leishmaniasis. Vaccine uptake differed significantly between regions (higher in the Algarve and the AML regions, Chi-square test, *χ*^2^ = 37.9, *df* = 4, *P* < 0.001). Over 80% of owners mentioned applying arthropod repellents or insecticides in the dog(s) and no statistically significant difference was found across NUTS2 regions (Chi-square test, *χ*^2^ = 7.9, *df* = 4, *P* = 0.096); spot-on was the most commonly identified (globally, 55.0%—and in every NUTS2 region, except for the Alentejo), followed by collar (40.9%); other types of applications are presented in Additional file 7: Fig. S3e. Use of these products all year round was reportedly common practice (62.8%) in all NUTS2 regions (Chi-square test, *χ*^2^ = 5.5, *df* = 4, *P* = 0.090). Regarding protection measures, 23.7% of donors reported having nets in some or all the windows/doors of their household–this practice was significantly more common in the Alentejo and the Algarve regions (Chi-square test, *χ*^2^ = 217.0, *df* = 4, *P* < 0.001). Practice of outdoor activities between dusk and dawn, on the other hand, was reported by 24.2% and less common in the Norte and AML regions (Chi-square test, *χ*^2^ = 30.2, *df* = 4, *P* < 0.001).

**Fig. 6:**
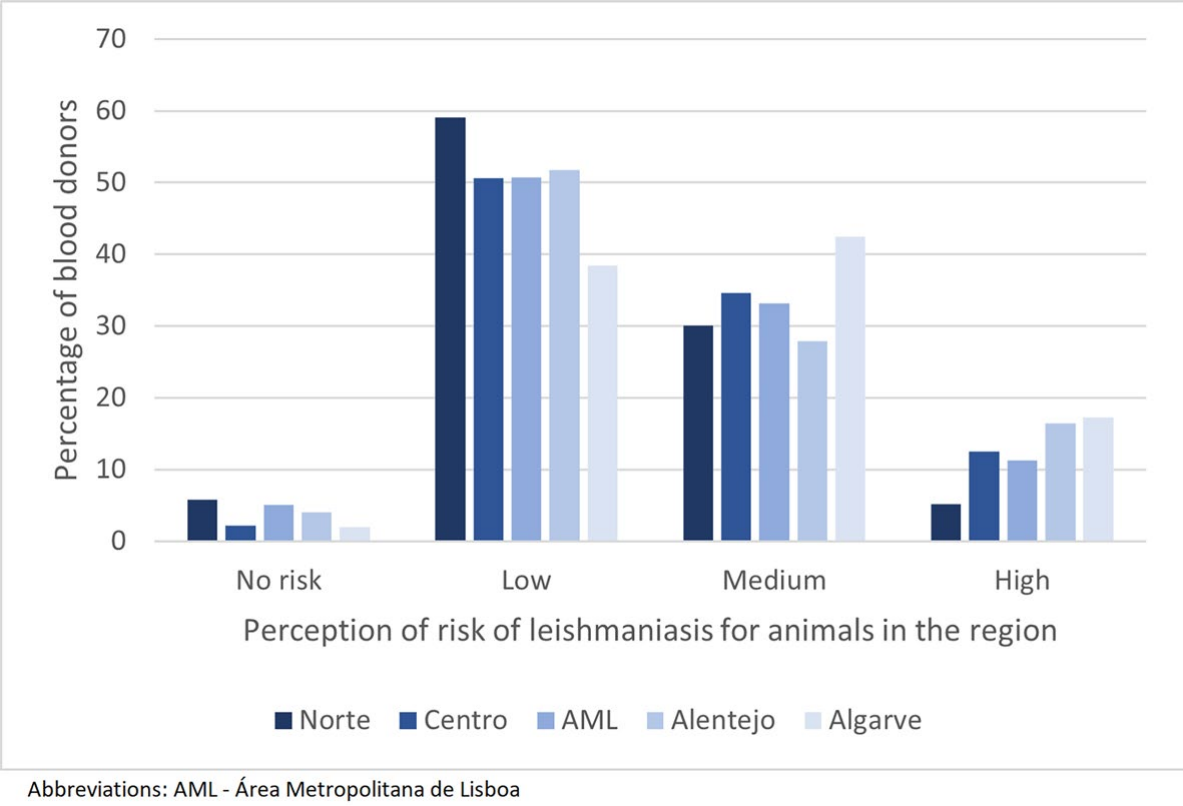
Perception of risk of leishmaniasis for animals in the region, according to NUTS2 region of residence

Regarding perceptions, classification of risk of leishmaniasis for animals was significantly different between donors of different NUTS2 regions (Chi-square test, *χ*^2^ = 38.4, *df* = 12, *P* < 0.001)—the Algarve was the region where “high” and “medium” risk were selected in the highest percentages, as presented in Fig. 6.

**Fig. 7:**
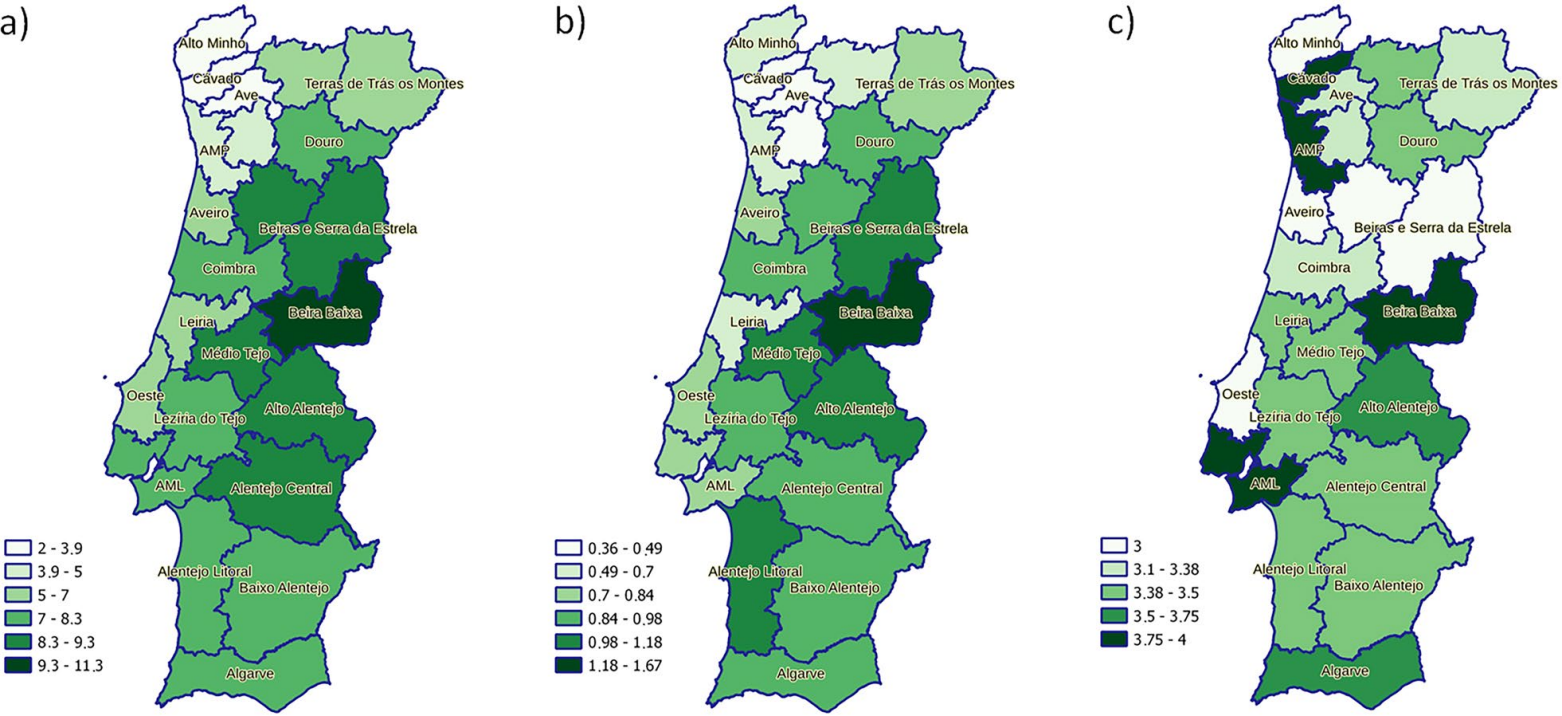
Distribution by NUTS3 region of **a** knowledge score (median), **b** perceptions score (mean), **c** practices score (median) (natural breaks were used to define categories)

Median values for individual *K* and Pra scores and mean values for individual Per score by NUTS2 and NUTS3 regions are presented in Table 4 and Fig. 7, respectively. The mean value was used to summarize the Per score by region since the median value was 0 in all but four NUTS3 regions. The median *K* score was lowest in the Norte region, especially in the coastal NUTS3 regions. Higher median *K* scores were obtained in the Centro and Alentejo regions, especially in the following NUTS3 regions (by descending order): Beira Baixa, Alto Alentejo, Médio Tejo, Alentejo Central, BSE, and Viseu Dão-Lafões. Higher mean Per scores largely overlapped with higher median *K* scores. On the other hand, median Pra scores were highest in the AML, Beira Baixa, and AMP, and lowest in the BSE, Viseu Dão-Lafões, Aveiro, Oeste, and Alto Minho regions. The distribution of individual *K*, Per, and Pra scores is represented in Additional file 6: Fig. S2.

**Table 4:** Distribution of knowledge (*K*), perceptions (Per), and practices (Pra) scores globally and by NUTS2 region

	Global	Norte	Centro	AML	Alentejo	Algarve	*P*-value
Total (*n*)	3763	1285	768	1005	463	242	
*K* score—median	7	4.5	8	8	8	7.6	< 0.001* (*H* = 115.5, *df* = 4)
(IQR)	(0–10.75)	(0–9.25)	(2–11.25)	(1–11)	(1–11)	(1–11.31)	
Per score—mean	0.78	0.55	0.93	0.84	0.95	0.98	< 0.001* (*H* = 86.6, *df* = 4)
(± SD)	(0–1.92)	(0–1.52)	(0–2.18)	(0–1.97)	(0–2.18)	(0–2.24)	
Pra score—median	3.5	3.5	3.25	4	3.5	3.62	< 0.001* (*H* = 64.2, *df* = 4)
(IQR)	(2.5–4.5)	(2.5–4.5)	(2.44–4)	(3–5)	(2.5–4.5)	(2.75–4.5)	

*AML* Área Metropolitana de Lisboa; *K* knowledge, *Per* perceptions, *Pra* practices, *IQR* interquartile range, *SD* standard deviation

*Statistically significant

### Associations between sociodemographic, knowledge, perceptions, and practices variables and asymptomatic infection

In univariate analysis, residing in the Centro region (considering the Alentejo as a reference category), male sex, not having previously heard of leishmaniasis, lower *K* score, and lower Per score were significantly associated with a positive serological result (Table 5). Higher seropositivity rates were also associated with those aged over 25 years; military, agriculture, and industry workers; and absence of mosquito nets in windows/doors, although these did not reach statistical significance. In multivariate analysis, factors associated with positive serological result were male sex (OR 1.75, 95% CI 1.30–2.38, *P* < 0.001), residing in the Centro region (OR 1.43, 95% CI 1.02–2.00, *P* = 0.039), and age over 25 years (OR 1.79, 95% CI 1.07–2.97, *P* = 0.026) (Table 6).

**Table 5:** Distribution of participants and positive serological results by category, for sociodemographic and knowledge, perceptions, and practices variables

Variables	Categories	Samples, % (*n*)	Seropositive donors, % (*n*)	*P*-value
Sex	Male	49.8 (1822/3655)	7.1 (129/1822)	< 0.001* (*χ*^2^ = 17.5, *df* = 1)
	Female	50.2 (1833/3665)	3.9 (72/1833)	
Age (years)	18–25	14.4 (513/3567)	3.3 (17/513)	0.127 (*χ*^2^ = 7.1, *df* = 4)
	26–35	20.6 (734/3567)	5.6 (41/734)	
	36–45	30.2 (1077/3567)	5.8 (62/1077)	
	46–55	24.6 (876/3567)	6.6 (58/876)	
	56–65	10.3 (367/3567)	6.3 (23/367)	
Level of education ^a^	1–4	1.9 (68/3565)	10.3 (7/68)	0.258 (FET = 5.3)
	5–9	16.8 (598/3565)	5.7 (34/598)	
	10–12	44.0 (1570/3565)	5.4 (85/1570)	
	Bachelor's	25.8 (920/3565)	5.9 (54/920)	
	MSc/PhD	11.5 (409/3565)	3.9 (16/409)	
Occupation^b^	Student	9.6 (279/2906)	3.2 (9/279)	
	Retired	1.9 (56/2906)	7.1 (4/56)	
	Unemployed	3.4 (100/2906)	8.0 (8/100)	
	0	1.9 (56/2906)	8.9 (5/56)	0.173 (*χ*^2^ = 4.4, *df* = 3)
	1–3	39.4 (1144/2906)	5.2 (60/1144)	
	4–5	25.7 (747/2906)	4.8 (36/747)	
	6–9	18.0 (524/2906)	7.1 (37/524)	
Travel abroad to endemic country ^c^ (< 2 years previously)	Yes	58.6 (439/749	5.2 (23/439)	0.739 (*χ*^2^ = 0.11, *df* = 1)
	No	41.4 (310/749)	5.8 (18/310)	
Type of parish of residence	Non-rural	58.3 (2133/3661)	5.5 (118/2133)	0.903 (*χ*^2^ = 0.02, *df* = 1)
	Rural	41.7 (1528/3661)	5.4 (83/1528)	
Had heard of leishmaniasis	Yes	72.4 (2638/3643)	4.9 (130/2638)	0.037* (*χ*^2^ = 4.3, *df* = 1)
	No/DK/CR	27.6 (1005/3643)	6.7 (67/1005)	
Animals affected by leishmaniasis nearby	Yes	11.7 (235/2017)	4.7 (11/235)	0.878 (*χ*^2^ = 0.02, *df* = 1)
	No/DK/CR	88.3 (1782/2017)	4.9 (87/1782)	
Regular contact with domestic animals	Yes	70.5 (2457/3486)	5.2 (127/2457)	0.419 (*χ*^2^ = 0.63, *df* = 1)
	No	29.5 (1029/3486)	5.8 (60/1029)	
Regular contact with wild animals	Yes	4.2 (139/3301)	2.9 (4/139)	0.180 (*χ*^2^ = 1.7, *df* = 1)
	No	95.8 (3162/3301)	5.4 (172/3162)	
Practice of outdoor activities during nighttime	Yes	24.1 (801/3321)	5.4 (43/801)	0.921 (*χ*^2^ = 0.01, *df* = 1)
	No	75.9 (2520/3321)	5.4 (137/2520)	
Use of nets in windows/doors	Yes (all/some)	23.8 (830/3481)	4.3 (36/830)	0.138 (*χ*^2^ = 2.2, *df* = 1)
	None	76.2 (2651/3481)	5.7 (150/2651)	
Ownership of dog(s)	Yes	48.1 (1727/3590)	5.5 (95/1727)	0.937 (*χ*^2^ = 0.01, *df* = 1)
	No	51.9 (1863/3590)	5.4 (101/1863)	
*K* score	≤ 7	50.7 (1858/3664)	6.4 (118/1858)	0.012* (*χ*^2^ = 6.2, *df* = 1)
	> 7	49.3 (1806/3664)	4.5 (81/1806)	
Per score	< 1	59.7 (2186/3664)	6.3 (137/2186)	0.006* (*χ*^2^ = 7.4, *df* = 1)
	≥ 1	40.3 (1478/3664)	4.2 (62/1478)	
Pra score	≤ 3,5	52.0 (1901/3664)	5.5 (104/1901)	0.937 (*χ*^2^ = 0.01, *df* = 1)
	> 3,5	48.0 (1763/3664)	5.4 (95/1763)	

*K* knowledge, *Per* perceptions, *Pra* practices, *MSc* Master of Science, *PhD* Doctor of Philosophy, *DK/CR* don't know/can't remember

*Statistically significant

^a^Categories refer to number of years completed of formal school education

^b^Category numbers refer to the numbers of the categories in the classification of European skills, competences, and occupations

^c^Albania, Brazil, Bulgaria, Cyprus, Eritrea, Ethiopia, Greece, India, Italy, Kenya, Malta, Nepal, Somalia, South Sudan, Spain, Sudan, Yemen

**Table 6:** Potential risk factors for asymptomatic *Leishmania* infection, according to logistic regression models to estimate crude and adjusted odds ratio values

Potential risk factor	Univariate			Multivariate		
	% in Sample	Crude OR	95% CI	Adjusted OR	95% CI	*P*-value
Older than 25 years	85.6	1.88	1.13–3.12	1.79	1.07–2.97	0.026*
Male sex	49.9	1.85	1.39–2.50	1.75	1.30–2.38	< 0.001*
Residing in Centro	20.4	1.37	0.99–1.91	1.43	1.02–2.00	0.039*
Had not heard of leishmaniasis	27.6	1.39	1.02–1.89	1.09	0.66–1.82	0.737
*K* score ≤ 7	50.7	1.39	1.04–1.85	1.14	0.68–1.89	0.621
Per score < 1	59.6	1.56	1.09–2.27	1.41	0.93–2.13	0.097
Constant				0.072		< 0.001
Hosmer–Lemeshow test				Sig. = 0.936		

*K* knowledge, *Per* perceptions, *OR* odds ratio, *CI* confidence interval

*Statistically significant

### Associations between sociodemographic variables and knowledge, perceptions, and practices

Univariate analysis of sociodemographic variables associated with higher than median *K*, Per, or Pra score is listed in Additional file 8: Table S5. In multivariate analysis, variables associated with above median K score were female sex (OR 1.23, 95% CI 1.05–1.43, *P* = 0.010), age between 25 and 40 years (OR 1.23, 95% CI 1.04–1.44, *P* = 0.013), residing outside the Norte region (OR 1.85, 95% CI 1.56–2.17, *P* < 0.001), ownership of dogs (OR 2.03, 95% CI 1.74–2.38, *P* < 0.001), higher education (OR 1.85, 95% CI 1.54–2.24, *P* < 0.001) and working as a military, manager, professional or technician (OR 1.44, 95% CI 1.20–1.73, *P* < 0.001). Factors associated with above median Per score were above median K score (OR 25.66, 95% CI 20.57–32.02, *P* < 0.001), residing outside the Norte region (OR 1.41, 95% CI 1.14–1.72, *P* = 0.002) and ownership of dogs (OR 1.37, 95% CI 1.12–1.67, *P* = 0.002). Lastly, even though the multivariate logistic model for Pra score was not statistically satisfactory (HL: *P* < 0.05), suggested factors associated with above median Pra score as follows: lower than median Per score (OR 1.35, 95% CI 1.16–1.59, *P* < 0.001), residing outside the Centro region (OR 1.52, 95% CI 1.25–1.85, *P* < 0.001), in a non-rural parish (OR 1.64, 95% CI 1.41–1.92, *P* < 0.001), and working as a military, manager, professional or technician (OR 1.29, 95% CI 1.08–1.55, *P* = 0.005) (Table 7).

**Table 7:** Potential factors for above median *K*, Per, or Pra score, according to logistic regression models to estimate crude and adjusted odds ratio values

	Potential risk factor	Univariate			Multivariate		
		% in Sample	Crude OR	95% CI	Adjusted OR	95% CI	*P*-value
*K* > 7	Age 25–40 years	35.7	1.32	1.15–1.51	1.23	1.04–1.44	0.013*
	Female sex	50.2	1.27	1.12–1.44	1.23	1.05–1.43	0.010*
	Residing outside Norte	65.9	1.96	1.69–2.22	1.85	1.56–2.17	< 0.001*
	Ownership of dogs	48.1	1.89	1.66–2.15	2.03	1.74–2.38	< 0.001*
	Higher education ^a^	37.3	2.19	1.91–2.51	1.85	1.54–2.24	< 0.001*
	Profession group 0–3 ^b^	41.0	1.95	1.68–2.26	1.44	1.20–1.73	< 0.001*
	Constant				0.510		< 0.001
	Hosmer–Lemeshow test				Sig. = 0.568		
Per ≥ 1	Age 25–40 years	35.7	1.30	1.13–1.49	1.18	0.97–1.45	0.106
	Female sex	50.2	1.22	1.07–1.39	0.98	0.81–1.20	0.865
	Residing outside Norte	65.9	1.89	1.64–2.17	1.41	1.14–1.72	0.002*
	Ownership of dogs	48.1	1.88	1.65–2.15	1.37	1.12–1.67	0.002*
	Higher education ^a^	37.3	1.80	1.57–2.07	1.13	0.89–1.43	0.328
	Profession group 0–3 ^b^	41.0	1.51	1.30–1.75	0.88	0.70–1.12	0.297
	*K* score > 7	49.3	31.34	25.81–38.06	25.66	20.57–32.02	< 0.001*
	Constant				0.093		< 0.001
	Hosmer–Lemeshow test				Sig. = 0.379		
Pra > 3.5	Age 25–40 years	35.7	1.03	0.90–1.17	0.89	0.76–1.04	0.153
	Female sex	50.2	1.06	0.93–1.20	1.07	0.92–1.24	0.379
	Residing outside Centro	79.6	1.72	1.47–2.04	1.52	1.25–1.85	< 0.001*
	Non-rural parish	58.0	1.72	1.52–1.96	1.64	1.41–1.92	< 0.001*
	Higher education ^a^	37.3	1.40	1.22–1.60	1.17	0.97–1.41	0.094
	Profession group 0–3 ^b^	41.0	1.44	1.25–1.67	1.29	1.08–1.55	0.005*
	Per score < 1	59.8	1.23	1.09–1.41	1.35	1.16–1.59	< 0.001*
	Constant				1.244		0.007
	Hosmer–Lemeshow test				Sig. = 0.039		

*K* knowledge, *Per* perceptions, *Pra* practices, *OR* odds ratio, *CI* confidence interval

*Statistically significant

^a^Completed bachelor's degree or above

^b^Categories 0–3 in the classification of European Skills, Competences, and Occupations: armed forces, managers, professionals, technicians

## Discussion

This study represents the first nationwide *Leishmania* human seroprevalence study in Portugal. National true seroprevalence was estimated at 4.8%, with regional values ranging from 0.0 to 9.2%. Previous small, regional studies (AML and BSE regions) have shown seroprevalence of 0.0–2.0% in asymptomatic individuals [14, 15]. These studies not only used different serological techniques (IFAT and Direct Agglutination Test [DAT], respectively), but also analyzed different populations (dog owners and outpatients in a tertiary hospital, respectively). In neighboring Spain, regional seroprevalence studies in blood donors have shown values between 1.3 and 7.9% [9, 12, 31–34]. The range of values of true seroprevalence estimated in the present study was similar to these Spanish studies. Studies in the general population of other Mediterranean countries have reported similar findings in Italy [35, 36] and Greece [37], although in some regions of Italy [38] and Croatia [39] seroprevalence exceeded 10%. Of note, studies in which each blood sample was simultaneously tested for the presence of anti-*Leishmania* antibodies and *Leishmania* nucleic acids generally showed low agreement between test results [9]. This raises the question of the biological meaning of the detection of antibodies against *Leishmania*, probably representing very diverse infection status, from previous exposure and elimination of the parasite to latency with undetectability of DNA in peripheral cells and to active replication at low, non-symptom-inducing levels. Large prospective studies in South Asia found no increased risk of progression to disease in asymptomatic seropositive people in general, except when baseline titers were very high or when seroconversion was recent (documented during follow-up) [40]. The absence of clear markers of progression to disease implies a currently limited clinical value of individually testing asymptomatic people, although doing so before programmed start of immunosuppressive therapy could lead to increased clinical and analytical monitoring of positive patients and thus to earlier diagnosis and treatment in case of progression to overt disease. In this sense, the results of the present study could indicate a possible role of screening patients living in areas where exposure is expected to be higher, such as the Médio Tejo and BSE regions. Another question raised is the potential of transmission of *Leishmania* by asymptomatic infected individuals, by blood donation; even though viable parasites and *Leishmania* DNA have been identified in donated blood, the risk of transmission has been considered absent or extremely low, when leucodepletion of donated blood is performed, as is current practice in Portugal [41, 42].

However, no single test has been suggested as a standard for defining the asymptomatic status and some authors would recommend combining serology and polymerase chain reaction (PCR) [10]. Even so, the several serological tests available were developed for diagnosing VL and often produce conflicting results in asymptomatic people [43, 44]. In the present study, only one serological test was used systematically, for all samples, and only one sample was tested per participant; the 298 positive or borderline samples were tested simultaneously by IFAT and only one was considered positive by both techniques, although 117 had detectable antibodies but bellow cut-off for positivity (titers from 8 to 32), suggesting possible low-level exposure to *Leishmania*. These results were not used for statistical analysis, since no ELISA-negative samples were simultaneously tested by IFAT. It is worth mentioning that it was assumed the specificity of the ELISA kits used was 99% as reported by the manufacturer. Additional comparative studies showed specificity ≥ 98% of this test for diagnosing VL in the Mediterranean setting [45, 46], but it is likely that specificity for asymptomatic infection could not be directly extrapolated.

Despite the many caveats for the use of serology for individual determination of asymptomatic infection status, the detection of antibodies to the parasite can be relevant from a public health perspective, especially when comparing the findings between different regions and by crossing the results with distribution of human VL cases and evidence from *Leishmania* in mammal hosts and vectors, following a One Health approach [25].

In Portugal, data on human cases drawn from public health surveillance in the period from 2013 to 2018, inclusively [3, 47], show that the NUTS3 regions with the highest incidence of VL cases (over 0.1/100000/year) were (in decreasing order) Alto Tâmega (0.396), Algarve, Alto Alentejo, Baixo Alentejo, Viseu Dão-Lafões, BSE, and Médio Tejo (0.146). No cases were detected in the following regions: Aveiro, Leiria, Oeste, Ave, Cávado, and Alentejo Litoral. However, data gathered via this passive surveillance system is probably incomplete, as suggested by a study of a previous period (1999–2009), where only 38.6% of cases diagnosed in hospitals were reported to the national surveillance system [4]. Additionally, it is likely that differences in incidence also represent a non-homogeneous underreporting across regions, especially considering that leishmaniasis is a rare disease in Portugal, and in lesser populated regions, one additional case can represent an important increase in incidence. In the study focusing on 1999–2009, the highest incidence, based on hospital records, was described in the following regions: Douro, Baixo Alentejo, Médio Tejo, AML, BSE, Coimbra, and Viseu Dão-Lafões.

All in all, this evidence suggests that Médio Tejo and BSE are among the regions of highest incidence, and these were among the regions with the highest seroprevalence in the present study. In the Algarve region, an increasing trend in incidence is suggested, especially considering most human cases between 2013 and 2018 (70%) were reported to public health surveillance in 2017 and 2018 [3]. In the present study, seroprevalence in the Algarve region was intermediate, but the eastern half of the region was not sampled, and, in the western part, sampling was based exclusively on a single central collection point. Although the suburban areas of Lisboa are classically considered endemic foci of leishmaniasis [48], the present study revealed intermediate seroprevalence in the AML. It is important to note, however, that incidence is related not only to exposure to *Leishmania*, but also to the presence of risk factors for progression to disease, which include immunosuppression, such as HIV infection. The two regions with higher incidence of HIV infection in most years between 2009 and 2018 were the AML and the Algarve [49], which could help explain the discrepancy between incidence of VL and *Leishmania* seroprevalence, compared to other regions of the country.

In the Alentejo region, seroprevalence was low in all NUTS3 regions, in contrast to human incidence data and canine seroprevalence studies for the Alto and Baixo Alentejo [16, 50]. One possible explanation is that in Alto Alentejo, approximately half the municipalities were insufficiently or not at all sampled, possibly including the ones where previous human cases have been detected. In the Baixo Alentejo region, sampling was based mostly on a single central collection point, meaning in some municipalities only a minority of parishes were sampled.

On the other hand, in the Alto Minho and Aveiro regions, human VL cases diagnosed have been very scarce [4] and canine seroprevalence is consistently lower [16, 50] but human seroprevalence was among the highest. In these regions, a significant proportion of positive or borderline individuals were residents in one or a few groupings of a small number of contiguous parishes, possibly highlighting the very localized nature of *Leishmania* circulation, related to the limited mobility of the vector. This was probably also the case for the Ave, Leiria, and Oeste regions (Fig. 4). However, as internal travel within Portugal was not assessed in the present study, it cannot be ruled out that a seropositive result could be related to exposure in other parts of the country.

In any case, these results raise the possibility of new foci of exposure to *Leishmania* in coastal and northern areas of Portugal (i.e., Ílhavo, Ovar, and Viana do Castelo) as a consequence of the expansion of the parasite due to global warming, as described in northwestern Spanish provinces previously considered non-endemic for leishmaniasis. However, autochthonous cases were first reported in the 1990s and since then several studies have detected seropositive dogs in the region [51–53]. In Portugal, two nationwide canine surveys performed 12 years apart, seroprevalence increased globally and in most regions; interestingly, some districts in the northern region showed marked (about three times) increases such as Porto (from 3.2% in 2009 to 9.2% in 2021) and Braga (from 2.1% in 2009 to 6.9% in 2021).

In the present study, potential factors associated with seropositivity in multivariate analysis were male sex and older age. These findings are consistent with previous studies in the Mediterranean region [9, 13]. Older age could be related to cumulative exposure to *Leishmania*-infected sand flies [54]; no clear sociocultural explanation has been found in the present study for higher asymptomatic seroprevalence in the male sex. A higher incidence of disease in male sex has also been reported [55]; sex-related biological factors could play a role in the differential incidence of VL [56].

KPP results highlight that animal leishmaniasis is well-recognized among blood donors, especially in the central and southern parts of the country. Although dog owners had more frequently heard of the disease (78.2 vs. 68.0%), the percentage was still lower than described in previous studies in pet owners (83–91%) [20, 21], possibly because the proportion of participants from the Norte region was lower (26 vs. 34%) and also because these studies were performed in a veterinary clinic/hospital context. Human leishmaniasis, however, is less recognized (38.1%, very similar to a previous study [20]). Television was identified as the main source of information, possibly because leishmaniasis is mentioned in the insecticide and arthropod repellent advertisements broadcast. In many parts of the Norte region, where canine leishmaniasis is considered less endemic or non-endemic, it is expected, as seen in the present study, that television largely supersedes veterinary professionals as sources of information. Many respondents who had heard of leishmaniasis in animals were not able to describe any sign, and only approximately half of those who described them selected weight loss or skin lesions, which are commonly present in diseased dogs [57]. This could imply that tutors may theoretically recognize the risk of disease locally, but not be able to identify its signs early in their animals, leading to late seeking of veterinary attention. Leishmaniasis was mainly considered a dog’s disease, but some donors also mentioned that cats could be affected. This could represent either a growing awareness in the veterinary and general community of the risk of feline leishmaniasis, following increasing scientific evidence [58], or a possible confusion with toxoplasmosis (supported in some questionnaires by the indication of hearing about it during pregnancy). It is interesting to note that regions where a higher percentage of donors reported knowing animals with leishmaniasis in the surroundings relate to the districts where canine seroprevalence was shown to be higher, namely Alto Alentejo, Médio Tejo, BSE, and Algarve [16]. The vaccination rate against canine leishmaniasis was higher than suggested in previous studies (21.7% vs. 4.5–14.9%) [16, 20], although awareness and use of vaccines were significantly different between regions. Compared to previous studies, similar rates of insecticide/repellent use in dogs were reported (82.2% vs. 69.6–92%) when considering all types of substances, regardless of their activity against sand flies [16, 20]. However, no information was collected regarding either the chemical substances or whether the products were being applied according to the manufacturer’s recommendations.

In the present study, a higher level of knowledge was associated with higher perception of leishmaniasis risk, but this did not translate into more protective practices. In fact, a higher Pra score was significantly associated with a lower Per score. Perhaps this could be partially explained by the fact that one of the practices considered protective was no ownership of dogs, but being a dog owner was significantly associated with a higher perception of disease. Additionally, vaccination was the only practice assessed specifically targeting leishmaniasis, and it is expensive; other practices considered protective against leishmaniasis are also protective for other arthropod-transmitted infections, so that they could be the result of the perception of risk of these other specific infections or of arthropod-borne infection in general. This could also explain why, in multivariate analysis, there was no significant association between the detection of anti-*Leishmania* antibodies and lower *K*, Per, or Pra scores related to leishmaniasis. These findings cannot be extrapolated to the general Portuguese mainland population, since only people aged 18–65 were included and the profile of people who donate blood could be different from the age-matched general population in each region and between regions. In addition, the representativeness even of the blood donor population itself could have been affected by the difficulty in obtaining a truly probabilistic sample, due to logistic constraints in some regions. Another limitation of the present study is the source of data, collected by questionnaires, which could present significant biases, including socially desirable responding.

## Conclusions

A human *Leishmania* seroprevalence of 4.8% among blood donors in mainland Portugal was estimated in this study. Significant variations were found among regions. In some cases, the detection of positive or borderline results was very geographically restricted and could represent new foci of parasite circulation. These findings highlight the importance of following a One Health approach to tackling the challenges of climate change: investing in more detailed and localized studies of seroprevalence in human and canine populations, coupled with studies on the presence of *Leishmania* in phlebotomine sand flies. More consistent reporting of human and animal cases would also be vital to providing a complete picture of the national burden of disease.

Seropositivity was associated with male sex and older age, but not with lower KPP, as defined by the score developed for this study. Future studies should follow probabilistic sampling approaches but should include a broader healthy population.

Factors found to be significantly associated with higher levels of knowledge reinforce the need to educate older people and young adults, males, people from lower education levels, and those from less differentiated professional backgrounds. Knowledge regarding leishmaniasis is heterogeneous in the country, and education programs should target areas where emergence of this zoonosis is expected. Education programs could greatly impact canine and human health if they were directed towards people living in some of the coastal subregions of the NUTS2 Norte and Centro regions, where canine seroprevalence seems to be increasing and human seroprevalence was high. These programs should emphasize the practices associated with effectively lowering the risk of both animal and human infection; veterinarians and physicians could likely play a determinant role in potentiating perceptions and practices in pet owners and immunocompromised individuals, respectively.

## Supplementary Information

**Additional file 1: Table S1**. List of NUTS (Nomenclature of Territorial Units for Statistics) 2 and NUTS3 regions in Continental Portugal.

**Additional file 2: Figure S1.** Questionnaire about sociodemographic aspects and knowledge, perceptions, and practices regarding leishmaniasis.

**Additional file 3: Table S2.** Protocol implemented for scoring knowledge, perceptions, and practices of blood donors, according to the answers provided in the questionnaire.

**Additional file 4: Table S3.** Sociodemographic characteristics of the participants, globally and by NUTS (Nomenclature of Territorial Units for Statistics) 3 region.

**Additional file 5: Table S4.** Answers to knowledge, perceptions, and practices questions, globally and by NUTS (Nomenclature of Territorial Units for Statistics) 3 region.

**Additional file 6: Figure S2.** Distribution of individual: (a) Knowledge scores; (b) Perceptions scores; (c) Practices scores.

**Additional file 7: Figure S3**. Percentage of blood donors choosing different: (a) sources of information about leishmaniasis; (b) main routes of transmission of leishmaniasis; (c) most frequent clinical signs in animals; (d) most commonly affected organs in humans; (e) types of repellent/insecticide product used in own dog(s).

**Additional file 8: Table S5**. Distribution of participants and *K*, Per and Pra scores by category, for sociodemographic variables.

## Data Availability

The datasets generated and analyzed during the current study are not publicly available due to confidentiality commitment with the participants, as stated in the consent declaration, but are available from the corresponding author on reasonable request.
