# Supplementary material for: Prevalence of asymptomatic Leishmania infection and knowledge, perceptions, and practices in blood donors in mainland Portugal
Source: Parasit Vectors. 2023 Oct 10;16:357. doi: 10.1186/s13071-023-05980-1 (PMC10563231; doi:10.1186/s13071-023-05980-1)
Supplement: Supplementary file 1 — Additional file 1: Table S1. List of NUTS (Nomenclature of Territorial Units for Statistics) 2 and NUTS3 regions in Continental Portugal. [file 13071_2023_5980_MOESM1_ESM.docx]

**Additional file 1: Table S1**

List of NUTS2 and NUTS3 regions in Continental Portugal

(NUTS – Nomenclature of Territorial Units for Statistics)

| **Code** | **NUTS1** | **Code** | **NUTS2** | **Code** | **NUTS3** |
| --- | --- | --- | --- | --- | --- |
| PT1 | Continente | PT11 | Norte | PT111 | Alto Minho |
|  |  |  |  | PT112 | Cávado |
|  |  |  |  | PT119 | Ave |
|  |  |  |  | PT11A | Área Metropolitana do Porto |
|  |  |  |  | PT11B | Alto Tâmega |
|  |  |  |  | PT11C | Tâmega e Sousa |
|  |  |  |  | PT11D | Douro |
|  |  |  |  | PT11E | [Terras de Trás-os-Montes](https://en.wikipedia.org/wiki/Terras_de_Tr%C3%A1s-os-Montes) |
|  |  | PT15 | Algarve | PT150 | Algarve |
|  |  | PT16 | Centro | PT16B | Oeste |
|  |  |  |  | PT16D | Região de Aveiro |
|  |  |  |  | PT16E | Região de Coimbra |
|  |  |  |  | PT16F | Região de Leiria |
|  |  |  |  | PT16G | Viseu Dão-Lafões |
|  |  |  |  | PT16H | Beira Baixa |
|  |  |  |  | PT16I | Médio Tejo |
|  |  |  |  | PT16J | Beiras e Serra da Estrela |
|  |  | PT17 | Área Metropolitana de Lisboa | PT170 | Área Metropolitana de Lisboa |
|  |  | PT18 | Alentejo | PT181 | Alentejo Litoral |
|  |  |  |  | PT184 | Baixo Alentejo |
|  |  |  |  | PT185 | Lezíria do Tejo |
|  |  |  |  | PT186 | Alto Alentejo |
|  |  |  |  | PT187 | Alentejo Central |
