# Supplementary material for: Prevalence of asymptomatic Leishmania infection and knowledge, perceptions, and practices in blood donors in mainland Portugal
Source: Parasit Vectors. 2023 Oct 10;16:357. doi: 10.1186/s13071-023-05980-1 (PMC10563231; doi:10.1186/s13071-023-05980-1)
Supplement: Supplementary file 2 — Additional file 2: Figure S1. Questionnaire about sociodemographic aspects and knowledge, perceptions, and practices regarding leishmaniasis. [file 13071_2023_5980_MOESM2_ESM.pdf]

24. Do you have pet animals? (Please, select all options that apply)

Yes, dog(s)		Yes, other(s): __________		No	

*=> If you only selected "**Yes, other(s)**" or "**No**", please proceed to question number **28***

25. Do(es) your dog(s) spend time outdoors, between sunset and sunrise?

Yes		No		Don't know/Can't remember	

*=> If you selected "**Yes**", please specify where* (Please, select all options bellow that apply):

Garden/backyard		Park		Other(s): __________
Street/road		Forest/bush		

26. Do(es) your dog(s) use any insecticide or insect repellent product?

Yes		No		Don't know/Can't remember	

*=> If you selected "**Yes**", please specify* (Please, select all options bellow that apply):

26.1. What type of product(s)?	Collar		Pipette		Spray	
	Shampoo		Pills		Others	
26.2. What time(s) of the year?	All year round		Summer		Spring	
	Autumn		Winter			

27. Is/Are your dog(s) regularly seen by a veterinarian?

Yes		No		Don't know/Can't remember	

*=> If you selected "**Yes**", please specify how often:*

Every two years		Once a year		More than once a year		Don't know/Can't remember	

28. What is:

28.1. Your age?	__________years				
28.2. Your sex?	Male		Female		No answer
28.3. The zone where you've been living in the last month?	Town Hall (Concelho): ____________________<br>Parish (Freguesia): ____________________				
28.4. Your current job?	__________				

29. What is your level of education? (Please, select the highest level of studies you have completed)

Primary school (1st-4th)		Basic school (5th-9th)		High school (10th-12th)	
Bachelor's		Master's/PhD		None of the previous	

30. In the last 2 years, have you been living or traveling abroad from Portugal?

Yes		*In which country/countries?* __________
No		
Don't know/Can't remember		

This questionnaire ends here. We thank you for your participation.

UNIVERSIDADE NOVA DE LISBOA
INSTITUTO DE HIGIENE E MEDICINA TROPICAL DESDE 1902
GHTM
FCT Fundação para a Ciência e a Tecnologia
IPST Instituto Português do Sangue e da Transplantação, IP

Código de colheita

Colar aqui

Questionnaire on leishmaniasis

Please answer all the following questions, marking with an "X" the option(s) you consider more appropriate or writing down a short answer in the spaces provided.

1. Have you ever heard about leishmaniasis?

Yes		No		Don't know/Can't remember	

*=> If you selected "**No**" or "**Don't know/Can't remember**", please proceed to question number **22.***

2. In which context(s) have you heard about leishmaniasis? (Please, select all options that apply)

In school/university subjects		In my professional activity		In conversations with family and/or friends	
In a conversation with a veterinarian		In a conversation with a medical doctor		In journals/magazines	
In the television		In posters		Don't know/Can't remember	
On the internet		Through social media		Other(s): __________	

3. Leishmaniasis is a disease caused by (Please, select only one option)**:**

Chemicals		An infection		Don't know/Can't remember	
A nutritional problem		A genetic problem		Other(s): __________	

4. Leishmaniasis is mostly transmitted by: (Please, select all options that apply)

Mosquito bites		Sand fly bites		Flea bites	
Tick bites		Animal scratch or bite		Unprotected sex	
Exposure to polluted environments		Consumption of contaminated water/food		Don't know/Can't remember	
Direct contact with an infected person		Direct contact with infected animals		Other(s): __________	
Blood transfusion		From mother to child			

5. Does leishmaniasis affect animals?

Yes		No		Don't know/Can't remember	

*=> If you selected "**No**" or "**Don't know/Can't remember**", please proceed to question number **14.***

6. Which of the following symptoms can often be seen in animals with leishmaniasis? (Please, select <u>all</u> options that apply)

Coughing		Difficulty breathing		Don't know/Can't remember	
Convulsions		Skin lesions		Other(s): __________	
Vomiting and/or diarrhea		Changes in the nails			
Weight loss		Hair loss			

7. Leishmaniasis in animals (Please, answer each of the following lines):

7.1. Can be treated?	Yes		No		Don't know/Can't remember	
7.2. Can be fatal/lethal?	Yes		No		Don't know/Can't remember	
7.3. Can be prevented/avoided?	Yes		No		Don't know/Can't remember	

8. In Portugal, is there leishmaniasis in animals?

Yes		No		Don't know/Can't remember	

*=> If you selected "**No**" or "**Don't know/Can't remember**", please proceed to question number **13.***

9. Which of these animals is/are more affected by leishmaniasis in Portugal? (Please, select <u>all</u> options that apply)

Horses		Cattle, sheep, goats		Other wild animals	
Cats		Rabbits/hares		Don't know/Can't remember	
Dogs		Mice		Other(s): __________	

10. Has any animal in your house or close to your house ever had leishmaniasis?

Yes		No		Don't know/Can't remember	

11. What do you think is the risk of animals catching leishmaniasis in the area where you live?

None		Low		Medium		High		Don't know/Can't remember	

12. In Portugal, is there a vaccine against leishmaniasis for animals?

Yes		No		Don't know/Can't remember	

*=> If you selected "**Yes**", is/are your pet animal(s) vaccinated against leishmaniasis?*

Yes, every year		No		I don't have pet animals	
Yes, some years		Don't know/Can't remember			

13. Can animals catch leishmaniasis when they travel or live abroad from Portugal?

Yes		No		Don't know/Can't remember	

14. Does leishmaniasis affect people?

Yes		No		Don't know/Can't remember	

*=> If you selected "**No**" or "**Don't know/Can't remember**", please proceed to question number **22.***

15. Which part(s) of the human body is/are more frequently affected by leishmaniasis? (Please, select <u>all</u> options that apply)

Eyes		Brain		Other(s): __________
Skin		Heart and lungs		
Liver and spleen		Don't know/Can't remember		

16. Leishmaniasis in people (Please, answer each of the following lines):

16.1. Can be treated?	Yes		No		Don't know/Can't remember	
16.2. Can be fatal/lethal?	Yes		No		Don't know/Can't remember	
16.3. Can be prevented/avoided?	Yes		No		Don't know/Can't remember	

17. Can people catch leishmaniasis in Portugal?

Yes		No		Don't know/Can't remember	

*=> If you selected "**No**" or "**Don't know/Can't remember**", please proceed to question number **21.***

18. Has any doctor ever told you that you have/had leishmaniasis?

Yes		No		Don't know/Can't remember	

19. Has any person in your house or close to your house ever had leishmaniasis?

Yes		No		Don't know/Can't remember	

20. What do you think is your risk of catching leishmaniasis?

None		Low		Medium		High		Don't know/Not applicable	

21. Can people catch leishmaniasis when they travel or live abroad from Portugal?

Yes		No		Don't know/Can't remember	

22. In your daily life, do you have (Please, answer each of the following lines):

22.1. Regular contact with wild animals?	Yes		No		Don't know/Can't remember	
22.2. Regular contact with domestic animals?	Yes		No		Don't know/Can't remember	
22.3. Outdoor activities during the night?	Yes		No		Don't know/Can't remember	

23. Does your house have nets in the windows and/or doors?

Yes, in all of them		Yes, in some		None of them		Don't know/Can't remember	
