# Supplementary material for: Prevalence of asymptomatic Leishmania infection and knowledge, perceptions, and practices in blood donors in mainland Portugal
Source: Parasit Vectors. 2023 Oct 10;16:357. doi: 10.1186/s13071-023-05980-1 (PMC10563231; doi:10.1186/s13071-023-05980-1)
Supplement: Supplementary file 3 — Additional file 3: Table S2. Protocol implemented for scoring knowledge, perceptions, and practices of blood donors, according to the answers provided in the questionnaire. [file 13071_2023_5980_MOESM3_ESM.docx]

**Additional file 3: Table S2**

Protocol implemented for scoring Knowledge, Perceptions and Practices of blood donors, according to the answers provided in the questionnaire

1. Knowledge score

Total knowledge score for each donor obtained by adding individual question scores

Higher scores representing higher level of knowledge

(Minimum 0 – Maximum 19)

| Number of question | Description of question | Answer(s) selected | Score |
| --- | --- | --- | --- |
| 1 | Have you ever heard about leishmaniasis? | Yes | 1 |
|  |  | No  Don’t know/Can’t remember  No answer | 0 |
| 3 | Leishmaniasis is a disease caused by: | An infection | 1 |
|  |  | Chemicals  A nutritional problem  A genetic problem  Don’t know/Can’t remember  No answer | 0 |
| 4 | Leishmaniasis is mostly transmitted by: | Sand fly bites + 0 | 1 |
|  |  | Sand fly bites + other(s) | 0.75 |
|  |  | Mosquito bites ± other(s) (except Sand fly bites) | 0.5 |
|  |  | Flea bites OR Tick bites ± other(s) (except Sand fly bites AND Mosquito bites) | 0.25 |
|  |  | All other combinations  Don’t know/Can’t remember  No answer | 0 |
| 5 | Does leishmaniasis affect animals? | Yes | 1 |
|  |  | No  Don’t know/Can’t remember  No answer | 0 |
| 6 | Which of the following symptoms can often be seen in animals with leishmaniasis? | Selects all the following: Weight loss + Skin lesions + Changes in the nails + Hair loss + 0 | 1 |
|  |  | Selects all the above + other(s) | 0.75 |
|  |  | Selects some of the above + 0 | 0.5 |
|  |  | Selects some of the above + other(s) | 0.25 |
|  |  | Other(s)  Don’t know/Can’t remember  No answer | 0 |
| 7.1 | Leishmaniasis in animals can be treated? | Yes | 1 |
|  |  | No  Don’t know/Can’t remember  No answer | 0 |
| 7.2 | Leishmaniasis in animals can be fatal/lethal? | Yes | 1 |
|  |  | No  Don’t know/Can’t remember  No answer | 0 |
| 7.3 | Leishmaniasis in animals can be prevented/avoided? | Yes | 1 |
|  |  | No  Don’t know/Can’t remember  No answer | 0 |
| 8 | In Portugal, is there leishmaniasis in animals? | Yes | 1 |
|  |  | No  Don’t know/Can’t remember  No answer | 0 |
| 9 | Which of these animals is/are more affected by leishmaniasis in Portugal? | Dogs + 0 | 1 |
|  |  | Dogs + other(s) | 0.5 |
|  |  | Other(s)  Don’t know/Can’t remember  No answer | 0 |
| 12 | In Portugal, is there a vaccine against leishmaniasis for animals? | Yes | 1 |
|  |  | No  Don’t know/Can’t remember  No answer | 0 |
| 13 | Can animals catch leishmaniasis when they travel or live abroad from Portugal? | Yes | 1 |
|  |  | No  Don’t know/Can’t remember  No answer | 0 |
| 14 | Does leishmaniasis affect people? | Yes | 1 |
|  |  | No  Don’t know/Can’t remember  No answer | 0 |
| 15 | Which part(s) of the human body is/are more frequently affected by leishmaniasis? | Liver and spleen + Skin + 0 | 1 |
|  |  | Liver and spleen + Skin + other(s)  Liver and spleen + 0  Skin + 0 | 0.5 |
|  |  | Liver + other(s)  Skin + other(s)  Other(s)  Don’t know/Can’t remember  No answer | 0 |
| 16.1 | Leishmaniasis in people can be treated? | Yes | 1 |
|  |  | No  Don’t know/Can’t remember  No answer | 0 |
| 16.2 | Leishmaniasis in people can be fatal/lethal? | Yes | 1 |
|  |  | No  Don’t know/Can’t remember  No answer | 0 |
| 16.3 | Leishmaniasis in people can be prevented/avoided? | Yes | 1 |
|  |  | No  Don’t know/Can’t remember  No answer | 0 |
| 17 | Can people catch leishmaniasis in Portugal? | Yes | 1 |
|  |  | No  Don’t know/Can’t remember  No answer | 0 |
| 21 | Can people catch leishmaniasis when they travel or live abroad from Portugal? | Yes | 1 |
|  |  | No  Don’t know/Can’t remember  No answer | 0 |

1. Perceptions score

Total perceptions score for each donor obtained by adding individual question scores

Higher scores representing higher level of perception of risk

(Minimum 0 – Maximum 6)

| Number of question | Description of question | Answer(s) selected | Score |
| --- | --- | --- | --- |
| 11 | What do you think is the risk of animals catching leishmaniasis in the area where you live? | High | 3 |
|  |  | Medium | 2 |
|  |  | Low | 1 |
|  |  | None  Don’t know/Not applicable  No answer | 0 |
| 20 | What do you think is your risk of catching leishmaniasis? | High | 3 |
|  |  | Medium | 2 |
|  |  | Low | 1 |
|  |  | None  Don’t know/Not applicable  No answer | 0 |

1. Practices score

Total practices score for each donor obtained by adding individual question scores

Higher scores representing higher level of protective practices

(Minimum 0 – Maximum 6)

| Number of question | Description of question | Answer(s) selected | Score |
| --- | --- | --- | --- |
| 12 | Is/are your pet animal(s) vaccinated against leishmaniasis? | Yes, every year | 0.5 |
|  |  | Yes, some years | 0.25 |
|  |  | No  Don’t know/Can’t remember  I don’t have pet animals  No answer | 0 |
| 22.1 | In your daily life, do you have regular contact with wild  animals? | No | 1 |
|  |  | Yes  Don’t know/Can’t remember  No answer | 0 |
| 22.2 | In your daily life, do you have regular contact with domestic  animals? | No | 1 |
|  |  | Yes  Don’t know/Can’t remember  No answer | 0 |
| 22.3 | In your daily life, do you have outdoor activities during the  night? | No | 1 |
|  |  | Yes  Don’t know/Can’t remember  No answer | 0 |
| 23 | Does your house have nets in the windows and/or doors? | Yes, in all of them | 1 |
|  |  | Yes, in some | 0.5 |
|  |  | None of them  Don’t know/Can’t remember  No answer | 0 |
| 24 | Do you have pet animals? | Yes, other(s) + 0  No  No answer | 2 |
|  |  | Yes, dog(s) ± Yes, other(s) | 0 |
| 25 | Do(es) your dog(s) spend time outdoors, between sunset and sunrise? | No | 0.5 |
|  |  | Yes  Don’t know/Can’t remember  No answer | 0 |
| 26 | Do(es) your dog(s) use any insecticide or insect repellent product?  What time(s) of the year? | Yes + All year round  Yes + Summer + Spring + Autumn | 0.5 |
|  |  | Yes + combinations of seasons not listed above nor below  Yes + Not specified | 0.25 |
|  |  | Yes + Winter + 0  No  Don’t know/Can’t remember  No answer | 0 |
| 27 | Is/Are your dog(s) regularly seen by a veterinarian?  Please specify how often: | Yes + Once a year  Yes + More than once a year | 0.5 |
|  |  | Yes + Every two years  Yes + Don’t know/Can’t remember  Yes + Not specified | 0.25 |
|  |  | No  Don’t know/Can’t remember  No answer | 0 |
