# Supplementary figures and images for: Prevalence of asymptomatic Leishmania infection and knowledge, perceptions, and practices in blood donors in mainland Portugal
Source: Parasit Vectors. 2023 Oct 10;16:357. doi: 10.1186/s13071-023-05980-1 (PMC10563231; doi:10.1186/s13071-023-05980-1)

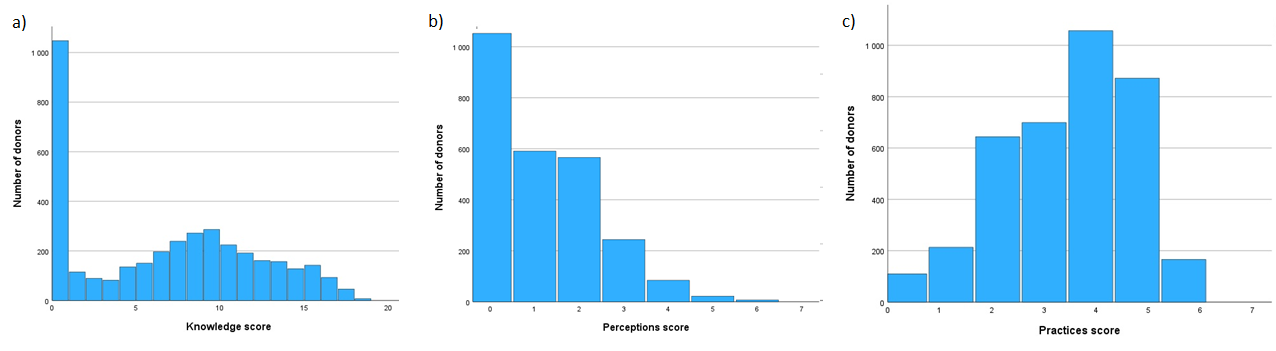

Supplement: Supplementary file 6 — Additional file 6: Figure S2. Distribution of individual: (a) Knowledge scores; (b) Perceptions scores; (c) Practices scores. [file 13071_2023_5980_MOESM6_ESM.png]

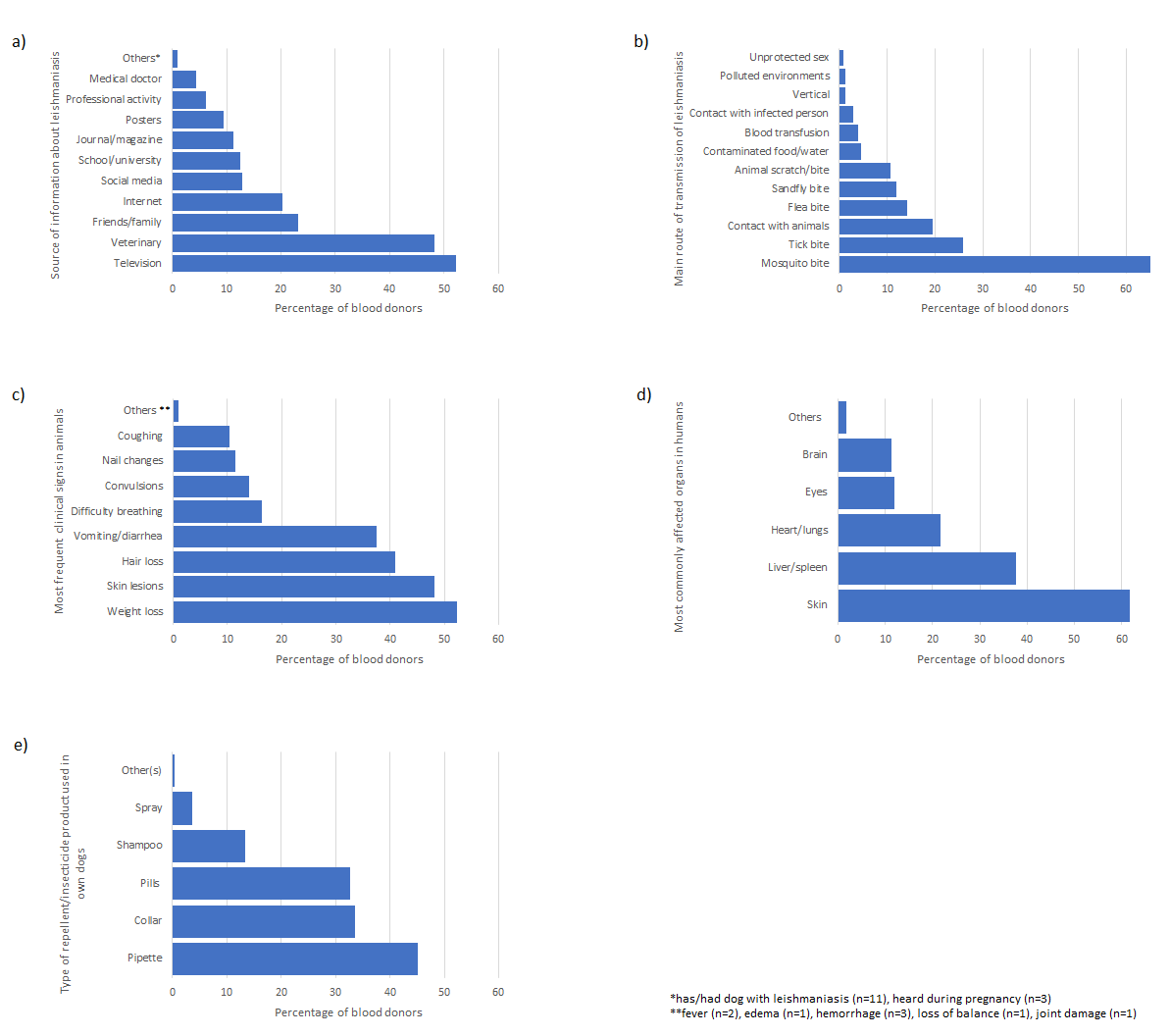

Supplement: Supplementary file 7 — Additional file 7: Figure S3. Percentage of blood donors choosing different: (a) sources of information about leishmaniasis; (b) main routes of transmission of leishmaniasis; (c) most frequent clinical signs in animals; (d) most commonly affected organs in humans; (e) types of repellent/insecticide product used in own dog(s). [file 13071_2023_5980_MOESM7_ESM.png]
