# Supplementary material for: Prevalence of asymptomatic Leishmania infection and knowledge, perceptions, and practices in blood donors in mainland Portugal
Source: Parasit Vectors. 2023 Oct 10;16:357. doi: 10.1186/s13071-023-05980-1 (PMC10563231; doi:10.1186/s13071-023-05980-1)
Supplement: Supplementary file 8 — Additional file 8: Table S5. Distribution of participants and K, Per and Pra scores by category, for sociodemographic variables. [file 13071_2023_5980_MOESM8_ESM.docx]

**Additional file 8: Table S5**

Distribution of participants and Knowledge, Perceptions and Practices scores by category, for sociodemographic variables.

| Variables | Categories | Samples, % *(n)* | K score >7, % (*n*) | | *p* value | | Per score ≥1, % (n) | | *p* value | | Pra score >3.5, % (n) | | *p* value | |  |  |
| --- | --- | --- | --- | --- | --- | --- | --- | --- | --- | --- | --- | --- | --- | --- | --- | --- |
| Sex | Male | 49.8  (1869/3750) | 46.3 (866/1869) | | **<0.001** | | 37.9 (708/1869) | | **0.003** | | 47.2 (883/1869) | | 0.391 | |  |  |
|  | Female | 50.2  (1881/3750) | 52.3 (984/1881) | |  |  | 42.7 (803/1881) | |  |  | 48.6 (915/1881) | |  |  |  |  |
| Age (y) | 18-25 | 14.6  (534/3662) | 49.4 (264/534) | | **0.021** | | 37.8 (202/534) | | **0.019** | | 42.7 (228/534) | | **0.045** | |  |  |
|  | 26-35 | 20.6  (756/3662) | 54.0 (408/756) | |  |  | 43.8 (331/756) | |  |  | 49.7 (376/756) | |  |  |  |  |
|  | 36-45 | 30.3  (1109/3662) | 49.5 (549/1109) | |  |  | 42.3 (469/1109) | |  |  | 50.5 (560/1109) | |  |  |  |  |
|  | 46-55 | 24.4  (895/3662) | 48.2 (431/895) | |  |  | 37.4 (335/895) | |  |  | 48.3 (432/895) | |  |  |  |  |
|  | 56-65 | 10.0  (368/3662) | 43.8 (161/368) | |  |  | 37.0 (136/368) | |  |  | 50.0 (184/368) | |  |  |  |  |
| Level of education* | 1-4 | 1.9 (69/3659) | 24.6 (17/69) | | **<0.001** | | 23.2 (16/69) | | **<0.001** | | 33.3 (23/69) | | **<0.001** | |  |  |
|  | 5-9 | 16.8  (615/3659) | 30.9  (190/615) | |  |  | 26.2 (161/615) | |  |  | 42.1 (259/615) | |  |  |  |  |
|  | 10-12 | 44.1 (1612/3659) | 48.2  (777/1612) | |  |  | 39.6 (638/1612) | |  |  | 47.1 (760/1612) | |  |  |  |  |
|  | Bachelor's | 25.8  (944/3659) | 61.1  (577/944) | |  |  | 48.7 (460/944) | |  |  | 52.5 (496/944) | |  |  |  |  |
|  | MSc/PhD | 11.5 (419/3659) | 64.4  (270/419) | |  |  | 52.3 (219/419) | |  |  | 56.3 (236/419) | |  |  |  |  |
| Occupation** | Student | 9.9  (295/2993) | 47.8 (141/295) | | **<0.001** | | 39.3 (116/295) | | **<0.001** | | 46.1 (136/295) | | **<0.001** | |  |  |
|  | Retired | 1.9 (56/2993) | 42.9 (24/56) | |  |  | 42.9 (24/56) | |  |  | 44.6 (25/56) | |  |  |  |  |
|  | Unemployed | 3.4  (101/2993) | 47.5 (48/101) | |  |  | 37.6 (38/101) | |  |  | 41.6 (42/101) | |  |  |  |  |
|  | 0 | 1.9 (57/2993) | 63.2 (36/57) | |  |  | 47.4 (27/57) | |  |  | 45.6 (26/57) | |  |  |  |  |
|  | 1-3 | 39.1 (1169/2993) | 59.7 (698/1169) | |  |  | 47.0 (550/1169) | |  |  | 54.3 (635/1169) | |  |  |  |  |
|  | 4-5 | 25.8  (773/2993) | 47.6 (368/773) | |  |  | 42.4 (328/773) | |  |  | 46.3 (358/773) | |  |  |  |  |
|  | 6-9 | 18.1 (542/2993) | 34.1 (185/542) | |  |  | 27.5 (149/542) | |  |  | 42.4 (230/542) | |  |  |  |  |
| Travel abroad to endemic country*** (<2y previosly) | Yes | 58.6 (450/768) | 54.4 (245/450) | | 0.119 | | 46.2 (208/450) | | 0.057 | | 52.4 (236/450) | | 0.205 | |  |  |
|  | No | 41.4 (318/768) | 48.7 (155/318) | |  |  | 39.3 (125/318) | |  |  | 47.8 (152/318) | |  |  |  |  |
| Type of parish | Non-rural | 58.0 (2178/3755) | 50.1 (1091/2178) | | 0.251 | | 40.7 (887/2178) | | 0.476 | | 53.6 (1167/2178) | | **<0.001** | |  |  |
|  | Rural | 42.0 (1577/3755) | 48.2 (760/1577) | |  |  | 39.6 (624/1577) | |  |  | 40.7 (633/1557) | |  |  |  |  |
| Ownership of dogs | Yes | 48.1  (1775/3687) | 58.3 (1034/1775) | | **<0.001** | | 48.8 (866/1775) | | **<0.001** | | NA | | NA | |  |  |
|  | No | 51.9  (1912/3687) | 42.5 (813/1912) | |  |  | 33.6 (643/1912) | |  |  | NA | |  |  |  |  |
| NUTS3 region | Norte | 34.1   (1285/3763) | 38.4 (494/1285) | | **<0.001** | | 30.5 (392/1285) | | **<0.001** | | 47.7 (613/1285) | | **<0.001** | |  |  |
|  | Centro | 20.4   (768/3763) | 53.1 (408/768) | |  |  | 45.4 (348/766) | |  |  | 37.2 (285/766) | |  |  |  |  |
|  | AML | 26.7  (1005/3763) | 56.0 (563/1005) | |  |  | 44.1 (443/1005) | |  |  | 57.0 (573/1005) | |  |  |  |  |
|  | Alentejo | 12.3   (463/3763) | 54.4 (252/463) | |  |  | 47.5 (220/463) | |  |  | 46.0 (213/463) | |  |  |  |  |
|  | Algarve | 6.4  (242/3763) | 56,6 (137/242) | |  |  | 45.9 (111/242) | |  |  | 50.0 (121/242) | |  |  |  |  |
|  |  |  | |  | |  | |  | |  | |  | |  | |  |
| *Categories refer to number of years completed of formal school education | | | | | |  | |  | |  | |  | |  | |  |
| **Category numbers refer to the numbers of the categories in the classification of European Skills, Competences, and Occupations | | | | | | | | | | | |  | |  | |  |
| ***Albania, Brazil, Bulgaria, Cyprus, Eritrea, Ethiopia, Greece, India, Italy, Kenya, Malta, Nepal, Somalia, South Sudan, Spain, Sudan, Yemen | | | | | | | | | | | | | |  | |  |
| Abbreviations: K - Knowledge; Per - Perceptions; Pra - Practices; y - years; MSc - Master of Science; PhD - Doctor of Phylosophy; NUTS - Nomenclature of territorial units for statistics; AML - Área Metropolitana de Lisboa | | | | | | | | | | | | | | | |  |
|  |  |  |  |  |  |  |  |  |  |  |  |  |  |  |  |  |
